# Spread and Molecular Characteristics of *Enterobacteriaceae* Carrying *fosA*-Like Genes from Farms in China

**DOI:** 10.1128/spectrum.00545-22

**Published:** 2022-07-19

**Authors:** Xiaoxiao Zhang, Mingxiang Ma, Yumeng Cheng, Yiqin Huang, Yuxiao Tan, Yunqiao Yang, Yajing Qian, Xin Zhong, Yujie Lu, Hongbin Si

**Affiliations:** a College of Animal Science and Technology, State Key Laboratory for Conservation and Utilization of Subtropical Agro-bioresources, Guangxi University, Nanning, China; University of Tennessee; Cornell University

**Keywords:** food animals, *Enterobacteriaceae*, *fosA*-like genes, fosfomycin resistance, transmission, farms, fosfomycin, genetic environments

## Abstract

In this study, we aimed to investigate the occurrence and molecular characteristics of fosfomycin-resistant *Enterobacteriaceae* isolates from pig, chicken and pigeon farms in Guangxi Province of China. A total of 200 fosfomycin-resistant strains were obtained from food animals and their surrounding environments, with the *fosA*, *fosA3*, and *fosA7.5* genes being detected in 26% (52/200), 10% (20/200), and 5% (10/200), respectively. Surprisingly, three *fosA7.5*-producing E. coli isolates were found to be concomitant with *fosA3*. Most of the *fosA*-like-gene-positive isolates were multidrug-resistant strains and consistently possessed *bla*_CTX-M-1/CTX-M-9_, *floR*, and *bla*_TEM_ genes. Only *fosA3* was successfully transferred to the recipient strains, and the 29 *fosA3*-carrying transconjugants exhibited high-level resistance to fosfomycin (MIC ≥ 512 μg/mL). Multilocus sequence typing (MLST) combined with enterobacterial repetitive intergenic consensus-PCR (ERIC-PCR) analyses indicated that *fosA3* or *fosA7.5* genes were spread by horizontal transfer as well as via clonal transmission between E. coli. We used the PCR mapping method to explore the genetic contexts of *fosA*-like genes, and two representative strains (fEc.1 and fEcg99-1) were fully sequenced. Six different genetic structures surrounding *fosA3* were detected and one infrequent context was discovered among the conjugable *fosA3*-positive E. coli isolates. The five genetic environments of *fosA* were identified and found to be highly similar to the partial sequence of transposon Tn*2921*. Furthermore, whole-genome sequencing (WGS) results showed that *fosA7.5* was colocalized with *mcr-3*, *bla*_CMY-63_, *sul3*, *tet*(A), *dfrA*, and a number of virulence-related factors on the same chromosomes of strains, and various insertion sequences (IS*3*/IS*L3*) were detected upstream or downstream of *fosA7.5*. The phylogenetic analysis revealed that both *fosA7.5*- and *fosA3*-carrying E. coli ST602 and *fosA7.5*-carrying E. coli ST2599 were closely related to E. coli isolates from humans, which may indicate that they pose a threat to human health.

**IMPORTANCE** Here, we report the widespread and complex genetic environments of *fosA*-like genes in animal-derived strains in China. The *fosA7.5* gene was identified in this study and was found to confer resistance to fosfomycin. The high prevalence of *fosA*-like genes in farms indicates that food animals serve as a potential reservoir for the resistance genes. This study also discovered that fosfomycin resistance genes were always associated with mobile elements, which would accelerate the transmission of *fosA*-like genes in strains. Importantly, E. coli ST602 and ST2599 carrying *fosA3* or *fosA7.5* from food animals had high similarity to E. coli isolates from humans, suggesting that *fosA*-like genes can be transmitted to humans through the food chain, thus posing a serious threat to public health. Therefore, the prevalence of *fosA*-like genes isolated from animals should be further monitored.

## INTRODUCTION

The wide spread of multidrug-resistant (MDR) Gram-negative bacteria, such as extended-spectrum-β-lactamase (ESBL)-producing *Enterobacteriaceae* and carbapenem-resistant *Enterobacteriaceae* (CRE), has resulted in fewer options for clinical treatment. In this case, fosfomycin, an older antibiotic agent, has garnered renewed interest and is considered a first-line antibiotic to treat infections caused by carbapenem-resistant and polymyxin-resistant bacteria (1). However, as the use of fosfomycin increased, so did the widespread dissemination of fosfomycin-resistant isolates in some countries. It has already been reported that fosfomycin resistance is relatively severe in China, with the resistance rates ranging from 25% to 50% (2–4). However, fosfomycin is still effective against ESBL-producing *Enterobacteriaceae* such as Salmonella, Escherichia coli, and Klebsiella pneumoniae in Europe, the Americas, and Africa (5).

Resistance to fosfomycin is primarily mediated by the expression of fosfomycin-modifying enzymes (FosA, FosB, and FosC), whereas the FosA enzyme encoded by chromosomes or plasmids is the most common in Gram-negative bacteria. To date, more than 10 *fosA-*like genes (*fosA1* to *fosA10*) have been identified, of which *fosA3* encodes the primary mechanism leading to fosfomycin resistance of E. coli and K. pneumoniae in China (6–8). Presently, *fosA3* is widely distributed among *Enterobacteriaceae* strains isolated from pets, pigs, chickens, and cows, as well as humans, although fosfomycin is not approved for use in animals in China (9–11). Furthermore, the coexistence of *fosA3* with other antibiotic resistance determinants (*bla*_CTX-M_, *bla*_TEM_, and *floR*) on plasmids has resulted in the emergence of fosfomycin-resistant strains in various countries around the world (12).

Previous research discovered that *fosA7* is mainly found on the chromosomes of Salmonella from various sources (human, cattle, sheep, and environment) (13). Subsequently, this gene was detected in different countries (14–16). In 2020, a study reported that the FosA identified in Escherichia coli differed from FosA7 protein, which was first reported in Salmonella, and its encoding gene was named *fosA7.5*^Q86E^ (17). At present, *fosA7.5* mainly exists in E. coli, and three variants of *fosA7.5* were discovered, of which *fosA7.5*^Q86E^ and *fosA*7.5^WT^ demonstrated resistance to fosfomycin. Moreover, the fosfomycin resistance gene *fosA* described in Serratia marcescens in 1980, which was the first *fosA*-like gene (namely, *fosA1*), also could confer high-level resistance to fosfomycin (18). However, limited information is available regarding the prevalence of *fosA* and *fosA7* among *Enterobacteriaceae* isolated from food animals, and no study has ever reported that *fosA3* and *fosA7.5* are coharbored in a single E. coli strain.

As a result, the strains containing *fosA*, *fosA3*, or *fosA7.5* from food animals and their environments were analyzed in this study to better understand their resistance phenotypes, plasmid replicon typing, genetic environments, and transmission characteristics. It provides a scientific foundation for future efforts to prevent the spread of fosfomycin resistance genes at the human-animal-environment interface.

## RESULTS

### Identification of fosfomycin resistance determinants and coexisting resistance genes.

In this study, a total of 200 fosfomycin-resistant *Enterobacteriaceae* isolates were obtained from the samples. Among these 200 strains, 82 were positive for *fosA*-like genes, and they came from chicken feces (*n* = 36), pig feces (*n* = 6), sewage from pig farms (*n* = 3), pig lungs (*n* = 4), pig nose (*n* = 4), pig mouth (*n* = 6), soil from pig farms (*n* = 4), soil from chicken farms (*n* = 4), pig anus (*n* = 1), pigeon (*n* = 12), and shells of chicken eggs (*n* = 2). Among the 82 *fosA*-like-gene-positive isolates, including 52 *fosA3*-positive E. coli isolates (26%; 52/200), 10 *fosA7.5*-positive E. coli isolates from pigeons (10%; 10/200), and 20 *fosA*-positive isolates (Enterobacter cloacae (*n* = 10), Escherichia hormaechei (*n* = 7) and Escherichia asburiae (*n* = 3) isolates) were also identified by 16S rRNA sequencing. Importantly, in the 10 *fosA7.5*-harboring E. coli isolates, three strains (KPg84, fEc.1, and ECg85) coharbored both *fosA7.5* and *fosA3*. However, *fosC2* and other fosfomycin resistance genes were not detected. Detailed information for the 82 strains is shown in Table S1 in the supplemental material.

**FIG 1:**
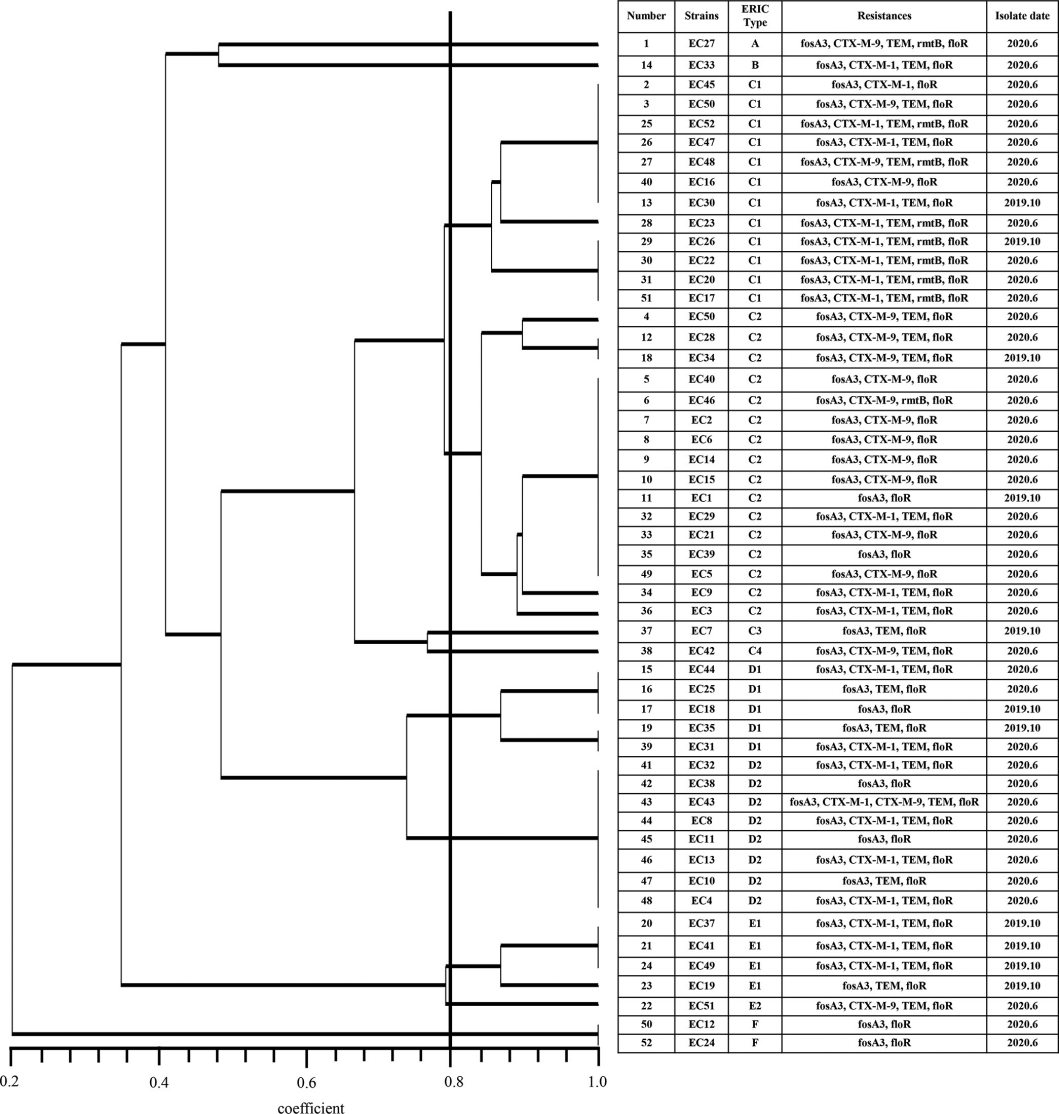
ERIC-PCR profiles of 52 *fosA3*-positive E. coli isolates.

The *fosA*/*fosA3*/*fosA7.5*-carrying *Enterobacteriaceae* isolates were also tested for the presence of other significant antibiotic resistance genes (ARGs). Screening for resistance genes confirmed that 40 of the 52 *fosA3*-positive E. coli isolates carried *bla*_CTX_-like resistance genes, and strain EC43 contained two different *bla*_CTX-M_ genes, including *bla*_CTX-M-1G_ and *bla*_CTX-M-9G_. In addition, 9 and 35 isolates harbored *rmtB* and *bla*_TEM_ genes, respectively, and all *fosA3*-carrying isolates were positive for *floR*. As a result, we identified the following gene combinations for *fosA3*: *fosA3*-*bla*_CTX-M-1_-*bla*_TEM_-*rmtB*-*floR* (*n* = 7), *fosA3*-*bla*_CTX-M-9_-*bla*_TEM_-*rmtB*-*floR* (*n* = 2) *fosA3*-*bla*_CTX-M-9_-*bla*_TEM_-*floR* (*n* = 5), *fosA3*-*bla*_CTX-M-1_-*bla*_TEM_-*floR* (*n* = 15), *fosA3*-*bla*_CTX-M-1_-*bla*_CTX-M-9_-*bla*_TEM_-*floR* (*n* = 1), *fosA3*-*floR* (*n* = 6), *fosA3*-*bla*_TEM_-*floR* (*n* = 6), *fosA3*-*bla*_CTX-M-1_-*floR* (*n* = 1), *fosA3*-*bla*_CTX-M-9_-*floR* (*n* = 8), and *fosA3*-*bla*_CTX-M-9_-*rmtB*-*floR* (*n* = 1) (Table 1; also, see Fig. 1). Except for strains ECg29 and EC315, all other *fosA7.5*-positive E. coli isolates carried *bla*_CTX-M_, *bla*_TEM_, and *floR* genes, and the most frequent gene profile was *fosA3*/*fosA7.5*-*bla*_CTX-M-1/CTX-M-9_-*floR*-*bla*_TEM_ (*n* = 8) (Table 2). However, most of the 20 *fosA*-positive strains showed a single-gene profile, and only one and four strains carried *bla*_NDM_- and *bla*_CTX_-like resistance genes, respectively (Table 2). The rates of *floR*, *bla*_TEM_, and *rmtB* genes were relatively low, at 20% (4/20), 10% (2/20), and 5% (1/20).

**TABLE 1:** Characterization of 29 conjugable *fosA3*-positive E. coli isolates

Strains	Context of *fosA3*^*a*^	Resistance profile^*b*^	Resistance genes
EC27	V	FFC, CHL, TET, CIP, FOS	*fosA3*, *bla*_CTX-M-9_, *bla*_TEM_, *rmtB*, *floR*
EC28	V	FFC, CHL, TET, CIP, FOS	*fosA3*, *bla*_CTX-M-9_, *bla*_TEM_, *floR*
EC29	I	CAZ, FFC, CHL, TET, CIP, FOS	*fosA3*, *bla*_CTX-M-1_, *bla*_TEM_, *floR*
EC30	I	CAZ, FFC, CHL, TET, TGC, FOS	*fosA3*, *bla*_CTX-M-1_, *bla*_TEM_, *floR*
EC31	II	CAZ, FFC, CHL, TET, FOS	*fosA3*, *bla*_CTX-M-1_, *bla*_TEM_, *floR*
EC32	II	CAZ, FFC, CHL, TET, CIP, AMK, COL, FOS	*fosA3*, *bla*_CTX-M-1_, *bla*_TEM_, *floR*
EC33	IV	CAZ, FFC, CHL, TET, CIP, FOS	*fosA3*, *bla*_CTX-M-1_, *bla*_TEM_, *floR*
EC34	V	FFC, CHL, TET, CIP, FOS	*fosA3*, *bla*_CTX-M-9_, *bla*_TEM_, *floR*
EC35	VI	FFC, CHL, TET, CIP, FOS	*fosA3*, *bla*_TEM_, *floR*
EC36	VI	CAZ, FFC, CHL, TET, CIP, AMK, FOS	*fosA3*, *bla*_CTX-M-1_, *bla*_TEM_, *rmtB*, *floR*
EC37	I	CAZ, FFC, CHL, TET, CIP, FOS	*fosA3*, *bla*_CTX-M-1_, *bla*_TEM_, *floR*
EC38	VI	FFC, TET, FOS	*fosA3*, *floR*
EC39	II	FFC, CHL, TET, CIP, TGC, FOS	*fosA3*, *bla*_TEM_, *floR*
EC40	IV	CAZ, FFC, CHL, TET, CIP, FOS	*fosA3*, *bla*_CTX-M-9_, *floR*
EC41	VI	CAZ, FFC, CHL, TET, CIP, TGC, FOS	*fosA3*, *bla*_CTX-M-1_, *bla*_TEM_, *floR*
EC42	V	FFC, CHL, TET, CIP, FOS	*fosA3*, *bla*_CTX-M-9_, *bla*_TEM_, *floR*
EC43	IV	CAZ, FFC, CHL, TET, CIP, COL, FOS	*fosA3*, *bla*_CTX-M-1_, *bla*_CTX-M-9_, *bla*_TEM_, *floR*
EC44	II	CAZ, FFC, CHL, TET, CIP, FOS	*fosA3*, *bla*_CTX-M-1_, *bla*_TEM_, *floR*
EC45	I	CAZ, FFC, CHL, TET, CIP, FOS	*fosA3*, *bla*_CTX-M-1_, *floR*
EC46	VI	CAZ, FFC, CHL, TET, CIP, TGC, FOS	*fosA3*, *bla*_CTX-M-9_, *rmtB*, *floR*
EC47	II	CAZ, FFC, CHL, TET, CIP, FOS	*fosA3*, *bla*_CTX-M-1_, *bla*_TEM_, *floR*
EC48	II	CAZ, FFC, CHL, TET, CIP, FOS	*fosA3*, *bla*_CTX-M-9_, *bla*_TEM_, *rmtB*, *floR*
EC49	VI	CAZ, FFC, CHL, TET, CIP, FOS	*fosA3*, *bla*_CTX-M-1_, *bla*_TEM_, *floR*
EC50	V	FFC, CHL, TET, CIP, FOS	*fosA3*, *bla*_CTX-M-9_, *bla*_TEM_, *floR*
EC51	VI	FFC, CHL, TET, CIP, TGC FOS	*fosA3*, *bla*_CTX-M-9_, *bla*_TEM_, *floR*
EC52	I	FFC, CHL, TET, AMK, TGC, FOS	*fosA3*, *bla*_CTX-M-1_, *bla*_TEM_, *rmtB*, *floR*
Kpg84	/	CAZ, FFC, CHL, TET, CIP, FOS	*fosA3*, *fosA7.5*, *bla*_CTX-M-1_, *floR*, *bla*_TEM_
fEc.1	III	CAZ, FFC, CHL, TET, CIP, FOS	*fosA3*, *fosA7.5*, *bla*_CTX-M-1_, *floR*, *bla*_TEM_
ECg85	/	CAZ, FFC, CHL, TET, CIP, FOS	*fosA3*, *fosA7.5*, *bla*_CTX-M-1_, *floR*, *bla*_TEM_

a /, the genetic environment of fosA3 was not detected.

b CAZ, ceftazidime; FFC, florfenicol; CHL, chloramphenicol; TET, tetracycline; CIP, ciprofloxacin; AMK, amikacin; COL, colistin; TGC, tigecycline; MEM, meropenem; FOS, fosfomycin.

**TABLE 2:** Characterization of 10 *fosA7.5*-positive isolates and 20 *fosA*-positive isolates

Strain	Resistance profile^*a*^	Resistance gene(s)
Kpg84	CAZ, FFC, CHL, TET, CIP, FOS	*fosA3*, *fosA7.5*, *bla*_CTX-M-1_, *floR*, *bla*_TEM_
fEc.1	CAZ, FFC, CHL, TET, CIP, FOS	*fosA3*, *fosA7.5*, *bla*_CTX-M-1_, *floR*, *bla*_TEM_
ECg85	CAZ, FFC, CHL, TET, CIP, FOS	*fosA3*, *fosA7.5*, *bla*_CTX-M-1_, *floR*, *bla*_TEM_
fEcg991	CAZ, FFC, CHL, TET, CIP, FOS	*fosA7.5*, *bla*_CTX-M-9_, *floR*, *bla*_TEM_
ECg29	CAZ, FFC, CHL, TET, CIP, FOS	*fosA7.5*, *floR*, *bla*_TEM_
ECg931	CAZ, FFC, CHL, TET, CIP, FOS	*fosA7.5*, *bla*_CTX-M-1_, *floR*, *bla*_TEM_
ECg932	CAZ, FFC, CHL, TET, CIP, FOS	*fosA7.5*, *bla*_CTX-M-1_, *bla*_CTX-M-9_, *floR*
ECg91	CAZ, FFC, CHL, TET, CIP, AMK, FOS	*fosA7.5*, *bla*_CTX-M-1_, *bla*_CTX-M-9_, *floR*
ECg933	CAZ, FFC, CHL, TET, CIP, FOS	*fosA7.5*, *bla*_CTX-M-1_, *bla*_CTX-M-9_, *floR*
EC315	FFC, FOS	*fosA7.5*, *floR*, *bla*_TEM_
20E.1	FFC, FOS, TET	*fosA*
20E.2	FFC, TGC, FOS, TET	*fosA*
EC2088	FFC, CHL, TET, TGC, FOS	*fosA*, *floR*
20E.4	FFC, CHL, TET, TGC, FOS	*fosA*, *floR*
20E.5	FFC, TET, TGC, FOS	*fosA*, *bla*_TEM_
20E.6	FFC, COL, TGC, FOS	*fosA*
20E.7	TET, COL, TGC, FOS	*fosA*
20E.8	TET, COL, TGC, FOS	*fosA*
20E.9	FFC, TET, COL, TGC, FOS	*fosA*, *bla*_CTX-M-9_
EC2098	FFC, CHL, TET, CIP, FOS	*fosA*, *floR*
20E.11	FFC, CHL, TET, FOS	*fosA*
KP20117	FFC, CHL, TET, COL, TGC, FOS	*fosA*
20E.13	CAZ, FFC, CHL, TET, CIP, COL, TGC, FOS	*fosA*, *bla*_CTX-M-9_
20E.14	CAZ, FFC, CHL, TET, CIP, COL, TGC, FOS	*fosA*, *bla*_CTX-M-9_
20E.15	CAZ, FFC, CHL, TET, CIP, AMK, FOS	*fosA*, *rmtB*
20E.16	CAZ, TET, CIP, COL, FOS	*fosA*
20E.17	CAZ, FFC, CHL, TET, COL, TGC, FOS	*fosA*, *bla*_TEM_, *floR*, *bla*_CTX-M-1_
20E.18	CAZ, TET, MEM, FOS	*fosA*, *bla*_NDM_
EC1928	FFC, CHL, TET, CIP, TGC, FOS	*fosA*
20E.20	FFC, COL, TGC, FOS	*fosA*

a CAZ, ceftazidime; FFC, florfenicol; CHL, chloramphenicol; TET, tetracycline; CIP, ciprofloxacin; AMK, amikacin; COL, colistin; TGC, tigecycline; MEM, meropenem; FOS, fosfomycin.

### Detection of antimicrobial resistance patterns.

**FIG 2:**
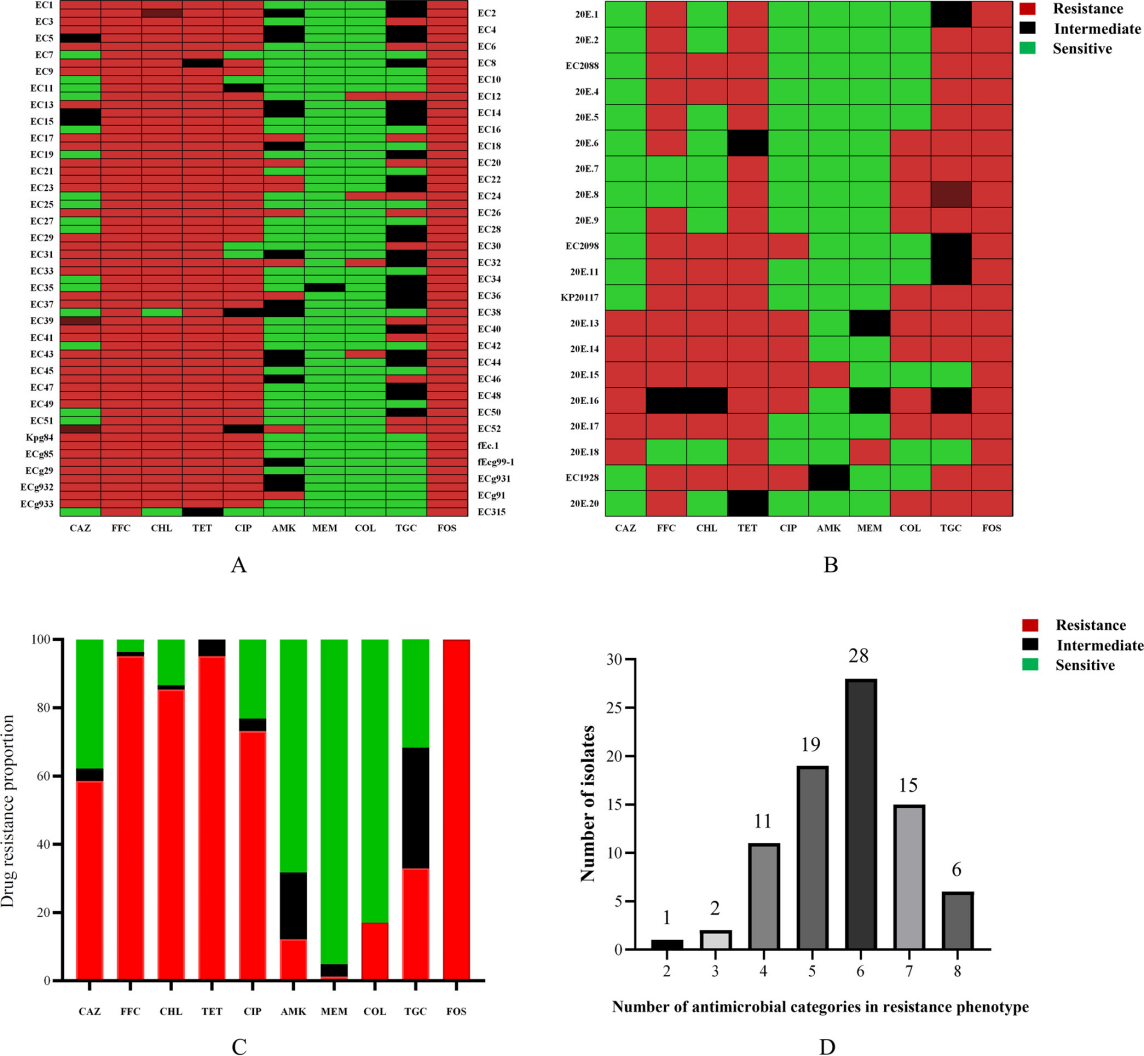
Analysis of the susceptibility results of 82 *Enterobacteriaceae* isolates with fosfomycin resistance for 13 antibiotics. (A and B) Drug resistance spectrum; (C) drug resistance proportion; (D) numbers of isolates with given numbers of antimicrobial categories in the resistance phenotypes.

In this study, 82 *Enterobacteriaceae* isolates containing *fosA*-like genes showed different degrees of resistance to 10 antimicrobial agents (Fig. 2A and B). Susceptibility testing indicated that all 82 strains were resistant to fosfomycin (100%; 82/82). These fosfomycin-resistant isolates also showed resistance to other antibiotics, such as ceftazidime (58.54%; 48/82), florfenicol (95.12%; 78/82), and chloramphenicol (85.37%; 70/82), tetracycline (90.24%; 74/82) and ciprofloxacin (73.17%; 60/82), and the resistance rates were all above 55%. However, only one and 10 strains were resistant to meropenem (1.22%) and amikacin (12.20%), respectively. It was also found that 16 strains (19.51%) exhibited intermediate resistance to amikacin (MIC = 4 μg/mL). Furthermore, several isolates were resistant to colistin (17.07%; 4/62) and tigecycline (20.97%; 14/82), with MICs at or above 2 μg/mL, and the resistant strains were mostly detected among *fosA*-positive isolates (Fig. 2C). Except for one strain that was only resistant to two antibiotics (fosfomycin and florfenicol), all 81 strains carrying *fosA*-like genes were multidrug-resistant strains (resistant to at least 3 classes of agents). According to the findings, 79 strains (98.75%) were resistant to 4 or more antibiotics, and six strains were resistant to all 8 antibiotics (Fig. 2D). The MICs of 82 strains are listed in Table S2 and S3.

### Conjugation experiments and plasmid analysis.

Among the 82 *fosA*/*fosA3*/*fosA7.5*-harboring isolates, 29 (35.37%; 29/82) were able to successfully transfer the fosfomycin resistance phenotype to E. coli recipient strain C600, and all transconjugants carried *fosA3*. For the three E. coli isolates coharboring both *fosA7.5* and *fosA*, only *fosA3* was successfully transferred from three donors to the recipient. Moreover, no *fosA* or *fosA7.5* transconjugants were acquired, indicating that these genes may be located on chromosomes or nonconjugative plasmids of strains. The MICs of 7 antimicrobial agents for 29 transconjugants are listed in Table 3, all of which were resistant to fosfomycin (MIC > 512 μg/mL). Furthermore, the 29 transconjugants showed resistance to ceftazidime (62.07%; 18/29), florfenicol (86.21%; 25/29), chloramphenicol (86.21%; 25/29), tetracycline (68.97%; 20/29), ciprofloxacin (17.24%; 5/29), and amikacin (10.34%; 3/29). It was found that 24 (82.76%) *fosA3*-carrying transconjugants were resistant to more than 4 antibiotics (Fig. S3). Enterobacterial repetitive intergenic consensus-PCR (ERIC-PCR) indicated that the bands of conjugants were consistent with E. coli C600, while showing differences with the donors (Fig. S4).

**TABLE 3:** MICs of 10 antimicrobial agents for the 29 *fosA3* transconjugants

Strain	MIC (μg/mL) of^*a*^:							
	CAZ	FFC	CHL	TET	CIP	AMK	RIF	FOS
EC27-T	<1	128	32	2	<1	<1	>1,000	>512
EC28-T	<1	256	128	64	<1	2	>1,000	>512
EC29-T	8	2	2	2	<1	<1	>1,000	>512
EC30-T	16	512	128	128	<1	2	>1,000	>512
EC31-T	4	256	64	32	<1	<1	>1,000	>512
EC32-T	16	4	2	2	<1	2	>1,000	>512
EC33-T	8	256	256	32	<1	<1	125	>512
EC34-T	<1	128	64	64	<1	<1	>1,000	>512
EC35-T	<1	256	128	128	8	2	>1,000	>512
EC36-T	8	256	64	64	<1	<1	>1,000	>512
EC37-T	16	512	128	256	<1	<1	>1,000	>512
EC38-T	<1	512	256	256	<1	<1	>1,000	>512
EC39-T	8	512	256	128	512	<1	1,000	>512
EC40-T	16	512	256	256	128	2	>1,000	>512
EC41-T	8	256	64	128	<1	2	>1,000	>512
EC42-T	<1	128	64	32	<1	2	>1,000	>512
EC43-T	16	2	2	2	<1	<1	>1,000	>512
EC44-T	8	4	2	64	<1	<1	>1,000	>512
EC45-T	8	256	128	16	<1	<1	>1,000	>512
EC46-T	8	256	64	512	64	2	>1,000	>512
EC47-T	<1	8	64	4	<1	8	>1,000	>512
EC48-T	<1	8	64	4	<1	16	>1,000	>512
EC49-T	16	512	256	256	<1	<1	>1,000	>512
EC50-T	<1	128	64	64	<1	<1	>1,000	>512
EC51-T	<1	256	128	64	<1	<1	>1,000	>512
EC52-T	8	128	128	64	2	8	>1,000	>512
fEc.1-T	8	256	128	2	<1	<1	>1,000	>512
Kpg84-T	8	256	64	<1	<1	<1	>1,000	>512
ECg85-T	8	256	128	2	<1	<1	>1,000	>512
C600	<1	<1	2	2	<1	<1	>1,000	<1

a CAZ, ceftazidime; FFC, florfenicol; CHL, chloramphenicol; TET, tetracycline; CIP, ciprofloxacin; AMK, amikacin; RIF, rifampicin; FOS, fosfomycin.

Except for three strains that carried both *fosA3* and *fosA7.5*, 26 conjugable *fosA3*-positive E. coli isolates included a total of 8 different plasmid replicon types, including Inc (I1, FIA, FIB, FII, K, HI1, HI2, N), and all of the strains contained 2 or more plasmid replicons (Table 4). The corresponding transconjugants also acquired different plasmid replicons; only IncFIB replicons were detected in two transconjugants (EC47-T and EC48-T), indicating that *fosA3* was located on Inc(FIB) plasmids. The results showed that multiple plasmids were transferred horizontally with the *fosA3* plasmids. Different from *fosA3*-positive isolates, seven plasmid replicons were detected in 10 *fosA7.5*-positive E. coli isolates (Table S4), including Inc (F_repB_, FIB, FII, I1, K, and Y). F_repB_ was discovered in all *fosA7.5*-positive E. coli strains, while IncY was found in six strains.

**TABLE 4:** Plasmid replicons of the 29 *fosA3*-positive E. coli and their transconjugants

Strain	Plasmid types	Transconjugant	Plasmid type(s)
EC27	HI2, FIB, FII, K	EC27-T	FIB, FII
EC28	FIB, FII, K	EC28-T	FIB, FII
EC29	I1, FIA, FIB, FII, K	EC29-T	I1, FIB, FII
EC30	I1, FIB, FII, K	EC30-T	FIB, FII
EC31	FIB, FII, K	EC31-T	FIB, FII
EC32	HI1, HI2, N, FIB, FII	EC32-T	N, FIB, FII
EC33	HI1, FIB, FII, K	EC33-T	FIB, FII
EC34	FIB, FII, K	EC34-T	FIB, FII
EC35	FIB, FII	EC35-T	FIB, FII
EC36	N, FIB, B, FII, K	EC36-T	N, FIB, FII
EC37	FIB, FII, K	EC37-T	FIB, FII
EC38	HI1, FIB, FII	EC38-T	FIB, FII
EC39	FIB, FII, K	EC39-T	FIB, FII
EC40	FIB, FII, K	EC40-T	FIB, FII
EC41	FIB, FII, K	EC41-T	FIB, FII
EC42	HI2, FIB, FII, K	EC42-T	FIB, FII
EC43	HI1, HI2, N, FIB, FII	EC43-T	N, FIB, FII
EC44	FIB, FII, K	EC44-T	FIB, FII
EC45	HI1, FIB, FII, K	EC45-T	FIB, FII
EC46	FIB, FII, K	EC46-T	FIB, FII
EC47	I1, N, FIB, B, FII, K	EC47-T	FIB
EC48	I1, N, FIB, B, FII, K	EC48-T	FIB
EC49	FIB, FII, K	EC49-T	FIB, FII
EC50	FIB, FII, K	EC50-T	FIB, FII
EC51	FIB, FII, K	EC51-T	FIB, FII
EC52	I1, FIB, FII, K	EC52-T	FIB, FII
Kpg84	F_repB_, FIB, FII, I1, K	Kpg84-T	I1, FIB, FII
fEc.1	F_repB_, FIB, FII, I1, K	Ecg87-T	I1, FIB, FII
ECg85	F_repB_, FIB, I1, FII, K	Kpg85-T	I1, FIB, FII
fEcg99-1	F_repB_, FIB, I1, Y, FII, K	None	None

### Strain typing (ERIC-PCR and MLST).

The genomic diversity analysis of 52 *fosA3*-positive strains and 10 *fosA7.5*-positive strains was analyzed by using the ERIC-PCR fingerprinting method. Among the 52 *fosA3*-positive E. coli isolates, the number of amplified bands ranged from 3 to 10, with sizes of 100 bp to 2,000 bp, and the genetic similarity was 20% to 100%. These isolates were divided into 6 main clusters (A to F) and 11 ERIC types, of which cluster C (C1 to C4) was the dominant genotype (59.62%; 31/52), and most of the strains in cluster C were derived from animal feces. Clusters A and B had the fewest strains, with only one strain in each cluster. The remaining five clusters (D to F) contained 13 (25%), 5 (9.62%) and 1 (3.85%) isolates, respectively (Fig. 1). MLST revealed a new sequence type (ST) and 15 known STs for the 29 conjugable *fosA3*-positive E. coli isolates, in which ST115 was the most common (*n* = 5), followed by ST156 (*n* = 4), ST7069 (*n* = 3), ST117 (*n* = 3), ST1196 (*n* = 2), ST23 (*n* = 2). Other STs were ST5229, ST683, ST202, ST224, ST410, ST1148, ST602, ST1468, and ST48, and each ST had one isolate. The two known STs (ST410 and ST23) belong to clonal complex 23 (CC23) and had only one difference in their *purA* alleles. The allele profiles of STs are provided in Table 5.

**TABLE 5:** The ST types and of conjugable *fosA3*-positive E. coli *and fosA7.5*-carrying isolates

No. of allele genes							ST^*a*^	Strain(s)
*adk*	*fumC*	*gyrB*	*icd*	*mdh*	*purA*	*recA*		
6	6	33	26	11	8	2	1196	EC27, EC42
6	4	14	16	24	8	14	115	EC28, EC34, EC35, EC44, EC50
112	11	5	12	8	8	6	7069	EC37, EC41, EC49
6	11	4	8	8	8	2	48	EC52
43	41	15	18	11	7	44	5229	EC29
20	45	41	43	5	32	2	117	EC32, EC38, EC43
6	4	12	1	20	13	7	23	EC47, EC48
6	4	127	16	24	8	6	683	EC30
6	4	12	1	20	18	7	410	EC36
6	4	33	16	11	8	6	224	EC33
6	95	3	18	11	7	14	1148	EC45
6	29	32	16	11	8	44	156	EC40, EC46, EC39, EC51
64	11	5	8	5	8	2	202	EC31
6	19	33	26	11	8	6	602	fEC.1
6	6	153	26	11	8	6	1468	ECg85
267	6	5	26	9	13	98	2599	fEcg99-1, ECg931, ECg933, ECg932, ECg91, ECg29
6	19	33	26	11	8	98	NA	Kpg84
653	19	270	26	11	8	7	7051	EC315

a NA, no ST type of the strain has been obtained.

**FIG 3:**
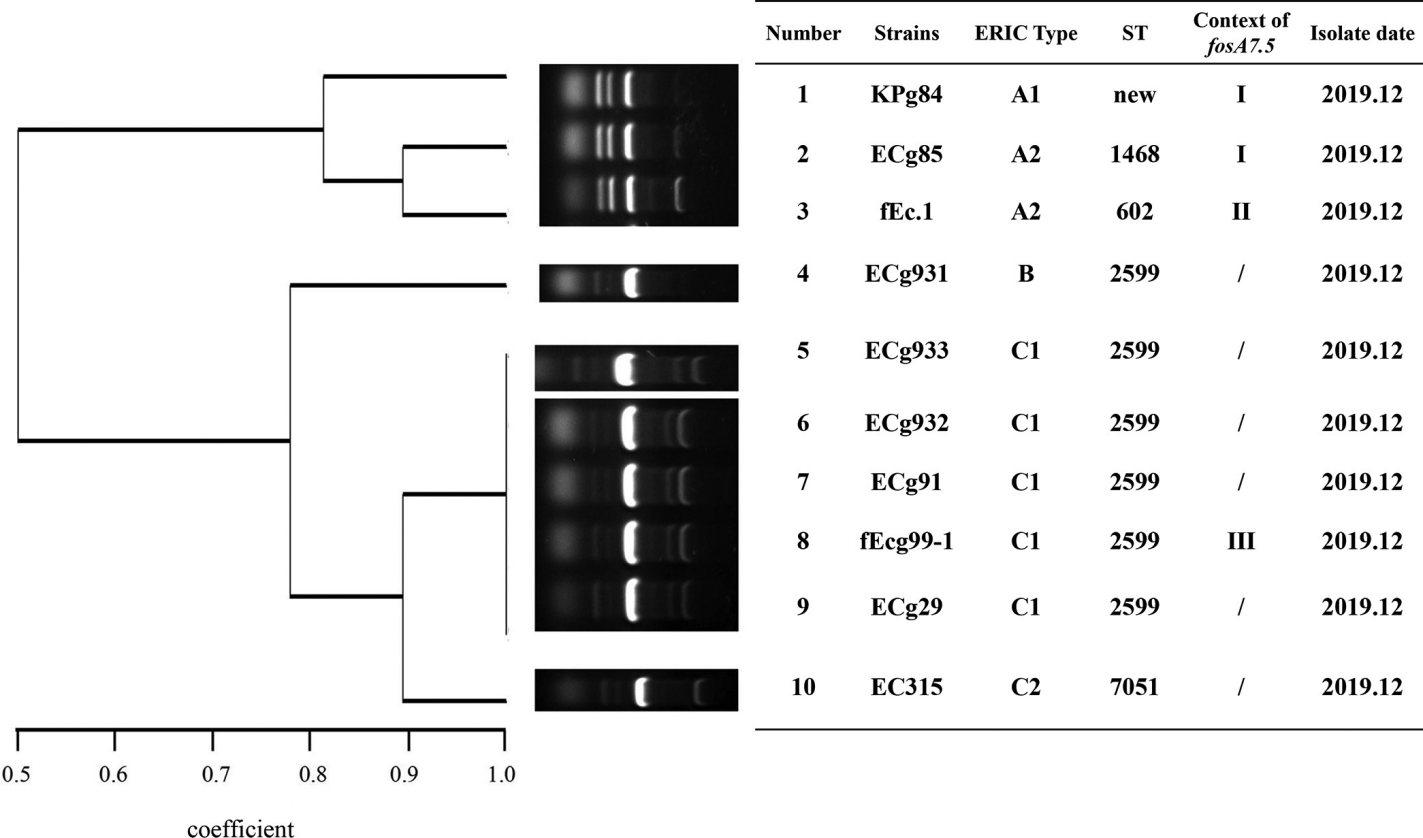
ERIC-PCR profiles of 10 *fosA7.5*-positive E. coli isolates.

MLST analysis showed that the 10 *fosA7.5*-positive E. coli isolates belonged to 4 STs (one ST1468, one ST602, six ST2599, and one ST7051), in which ST2599 was predominant. Analysis of the ERIC-PCR profiles showed that there were a total of 3 unique clusters (A, B, and C) and 5 ERIC types within 10 *fosA7.5*-carrying E. coli isolates. Except for strain ECg931, other ST2599 and ST7051 strains belonged to cluster C. Also, the three E. coli isolates carrying both *fosA3* and *fosA7.5* were classified as cluster A (Fig. 3). ERIC-PCR combined with MLST analyses indicated that the *fosA*-like genes were spread by horizontal transfer as well as via clonal transmission between *Enterobacteriaceae* isolates in the farms. The allele profiles of STs are provided in Table 5.

### Genetic background of *fosA* in E. cloacae and *E. hormaechei*.

**FIG 4:**
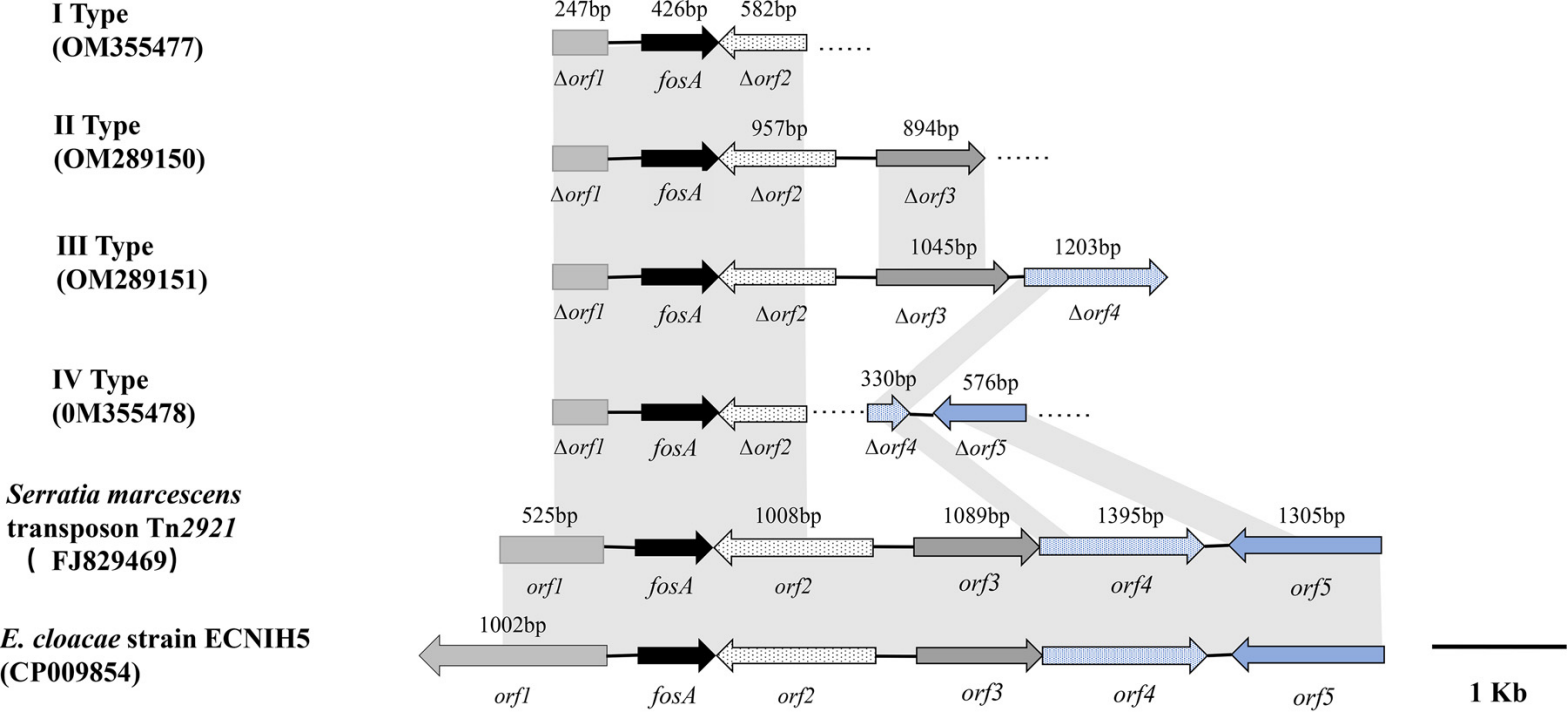
Genetic contexts of *fosA* in E. cloacae and *E. hormaechei*. *orf1, orf2, orf3, orf4*, and *orf5* encode part of the tryptophan tRNA synthetase, LacI family transcriptional regulator, glycosyl hydrolase family 2, MFS sugar transporter, and a restriction endonuclease. Shaded boxes between sequences indicate homologous regions (>90% sequence identity).

For the 17 *fosA*-positive isolates, four types of genetic contexts were identified by PCR mapping, all of which shared > 99% similarity with partial sequences in E. cloacae strain ECNIH5 (CP009854) and S. marcescens transposon Tn*2921* (FJ829469). The most common were type I (*n* = 7) and type III (*n* = 3), while the others were type II (*n* = 1) and type IV (*n* = 1). In all four types, a 247-bp length of amplicon in the upstream region of *fosA* was identical to the truncated tryptophan tRNA synthetase gene in E. cloacae strain ECNIH5 and transposon Tn*2921*. In the downstream region of *fosA*, we found four amplicons with lengths of 957 bp, 894/1,045 bp, 1,203 bp, and 576 bp encoding the LacI family transcriptional regulator, sugar-binding cellulose-like protein, MFS sugar transporter, and restriction endonuclease, respectively, which were similar to part of the sequence in transposon Tn*2921*. In type I, only a 582-bp length of amplicon encoding the LacI family transcriptional regulator was confirmed in the downstream region of *fosA* (Fig. 4).

### Genetic background of *fosA3* in E. coli isolates.

**FIG 5:**
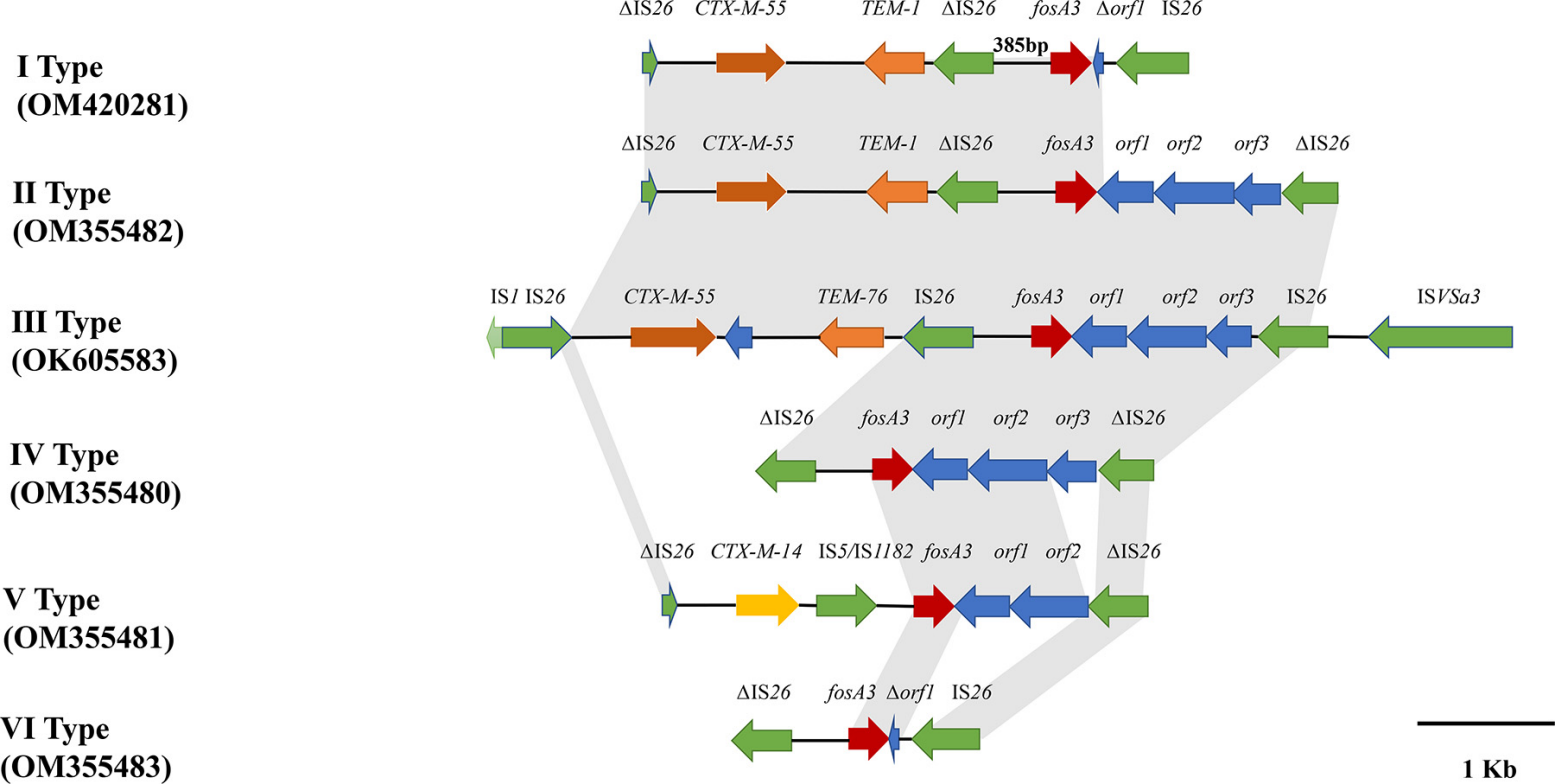
Genetic context of *fosA3* in E. coli. Arrows indicate the directions of transcription of the genes, and different genes are shown in different colors. Shaded boxes between sequences indicate homologous regions (>90% sequence identity). *orf1*, *orf2*, and *orf3* encode a hypothetical protein, a CadC-like protein, and a truncated TetR family transcriptional regulator.

PCR mapping was used to determine the regions adjacent to *fosA3* in 26 conjugable *fosA3*-positive E. coli isolates. Five different genetic contexts were identified, including type I (*n* = 4), type II (*n* = 6), type IV (*n* = 3), type V (*n* = 6), and type VI (*n* = 7) (Fig. 5). The *fosA3* gene was flanked by two IS*26* elements oriented in the opposite direction in 20 isolates. An IS*26* element was found to be located on downstream of *fosA3* in all isolates, and the lengths of the spacer regions between the 3′ end of *fosA3* and the IS*26* gene were variable (2,377 bp, 1,823 bp, and 707 bp). In type I, II, IV, and VI structures, the IS*26* element was located 385 bp upstream of *fosA3*. In addition, the extended-spectrum β-lactamase (ESBL) gene *bla*_CTX-M-55_ was frequently located upstream of *fosA3* in two genomic contexts (type I and type II), and a truncated IS*26* transposase determinant was identified upstream of *bla*_CTX-M-55_. The type V (*n* = 6) structure was from *bla*_CTX-M-14_-positive isolates; it was found that the IS*26* element upstream of *fosA3* was replaced by the ΔIS*26*-*bla*_CTX-M-14_-IS*5*/IS*1182*-*fosA3* structure, which was similar to that on plasmid on LWY24 (MT318677.1, chicken, E. coli) (Fig. 5).

**FIG 6:**
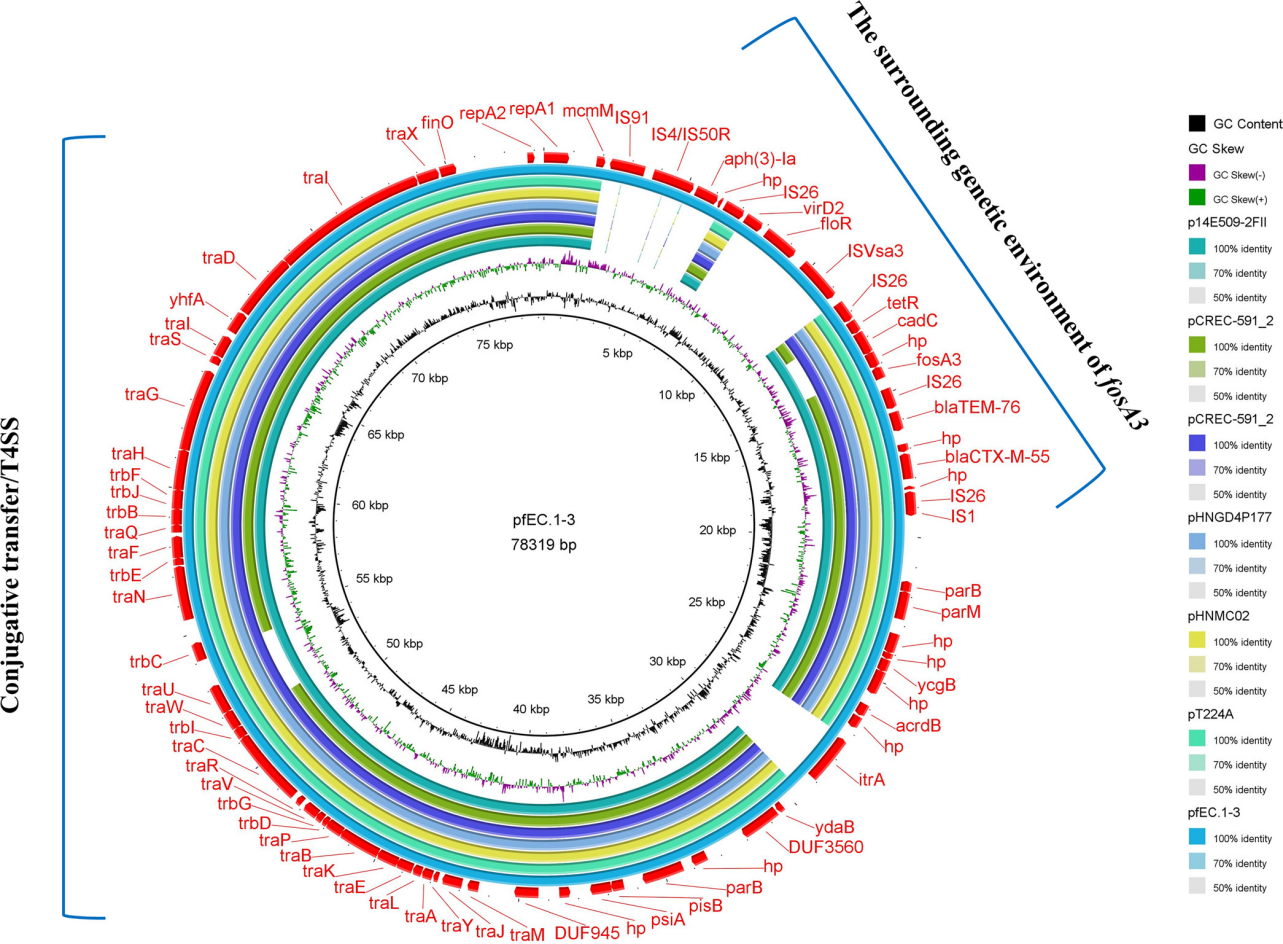
Comparative genomics analysis of IncFII plasmids carrying *fosA3*, the external ring represents the annotation of pfEC.1-3.

**FIG 7:**
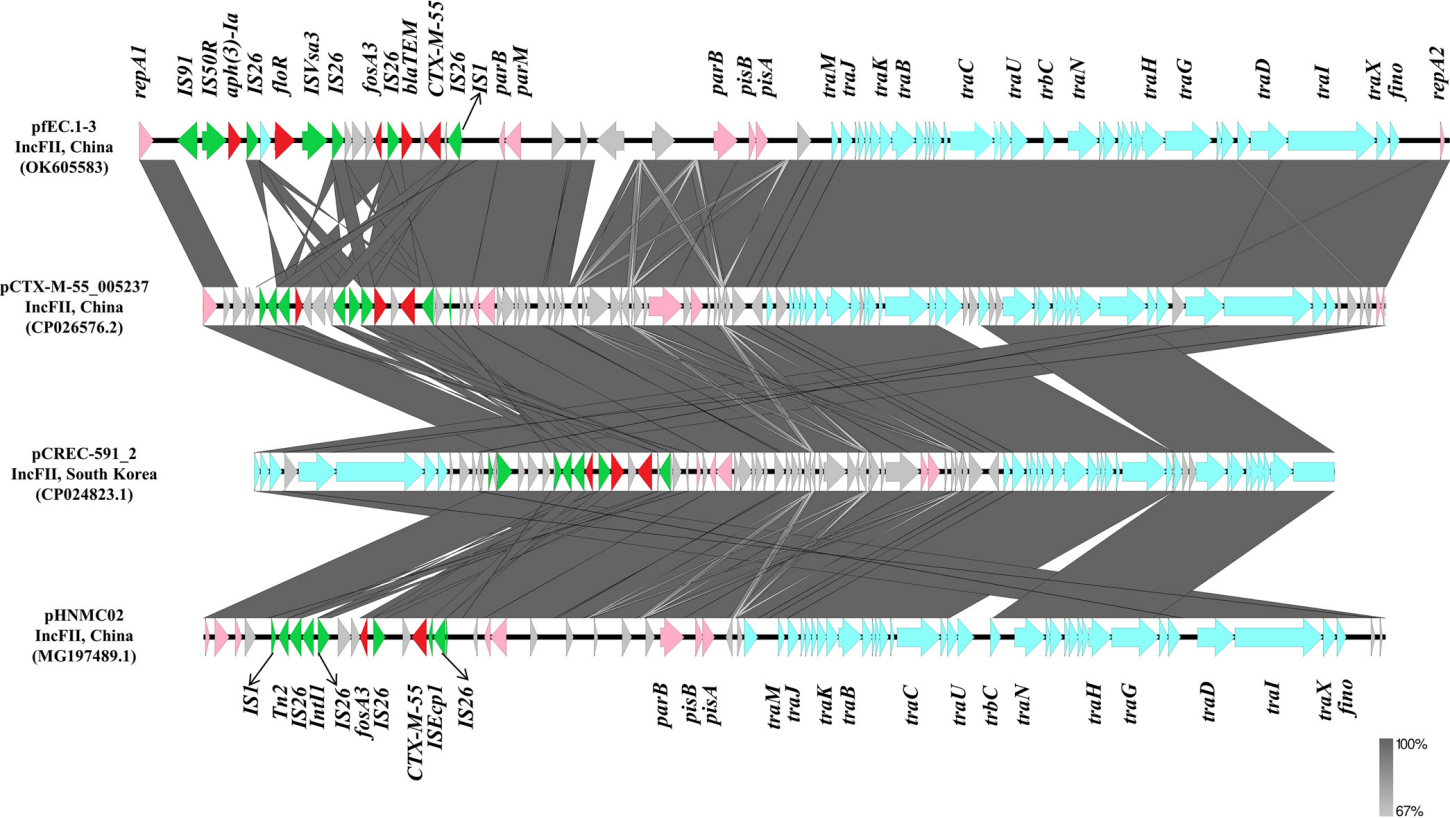
Comparison of the genetic environment of *fosA3* in pfEc.1-3 and other closely related IncFII plasmids.

One representative pigeon-derived E. coli isolate (fEC.1) carrying *fosA3* and *fosA7.5* was analyzed by whole-genome sequencing (WGS) and was identified as ST602. The *fosA3*-harboring plasmid was named pfEC.1-3 (OK605583), with a size of 78,319 bp. The plasmid belonged to the IncFII incompatibility group and contained a variable region responsible for *fosA3*. The two structures, IS*1*-IS*26*-*orf*-*bla*_CTX-M-55_-*orf*-*bla*_TEM_-76-IS*26*-*fosA3* and *fosA3*-*orf1*-*orf2*-*orf3*-IS*26*-IS*Vsa3*/IS*91*-*floR*-*aph(3′)-Ia*, were located upstream and downstream of *fosA3*, respectively, and were named type III. A BLAST search for pfEC.1-3 revealed highly homology (>90%) to six other known IncFII plasmids deposited in the GenBank database, which were p14E509-2FII (MN822125.1; China; human), pCREC-591_2 (CP024823.1; South Korea; human), pCTX-M-55_005237 (CP026576.2; China; human), pHNGD4P177 (MG197492.1; China; pig), pHNMC02 (MG197489.1; China; chicken) and pT224A (MW298658.1; Canada; dairy cow). All plasmids had backbone genes associated with IncFII plasmid replication (*repA1*/*A2*), conjugative transfer and the type IV secretion system (T4SS) (*tra* and *trb*), separation (*parM*), and maintenance of genetic stability (*stbA*) (Fig. 6). However, the variations between these plasmids resulted from insertion sequences (IS*26*, IS*4*, and IS*91*), integrase (IntI), and resistance [*floR* and *aph(3′)-Ia*] genes around the *fosA3* gene (Fig. 7).

### Phylogenetic analysis of fEC.1.

**FIG 8:**
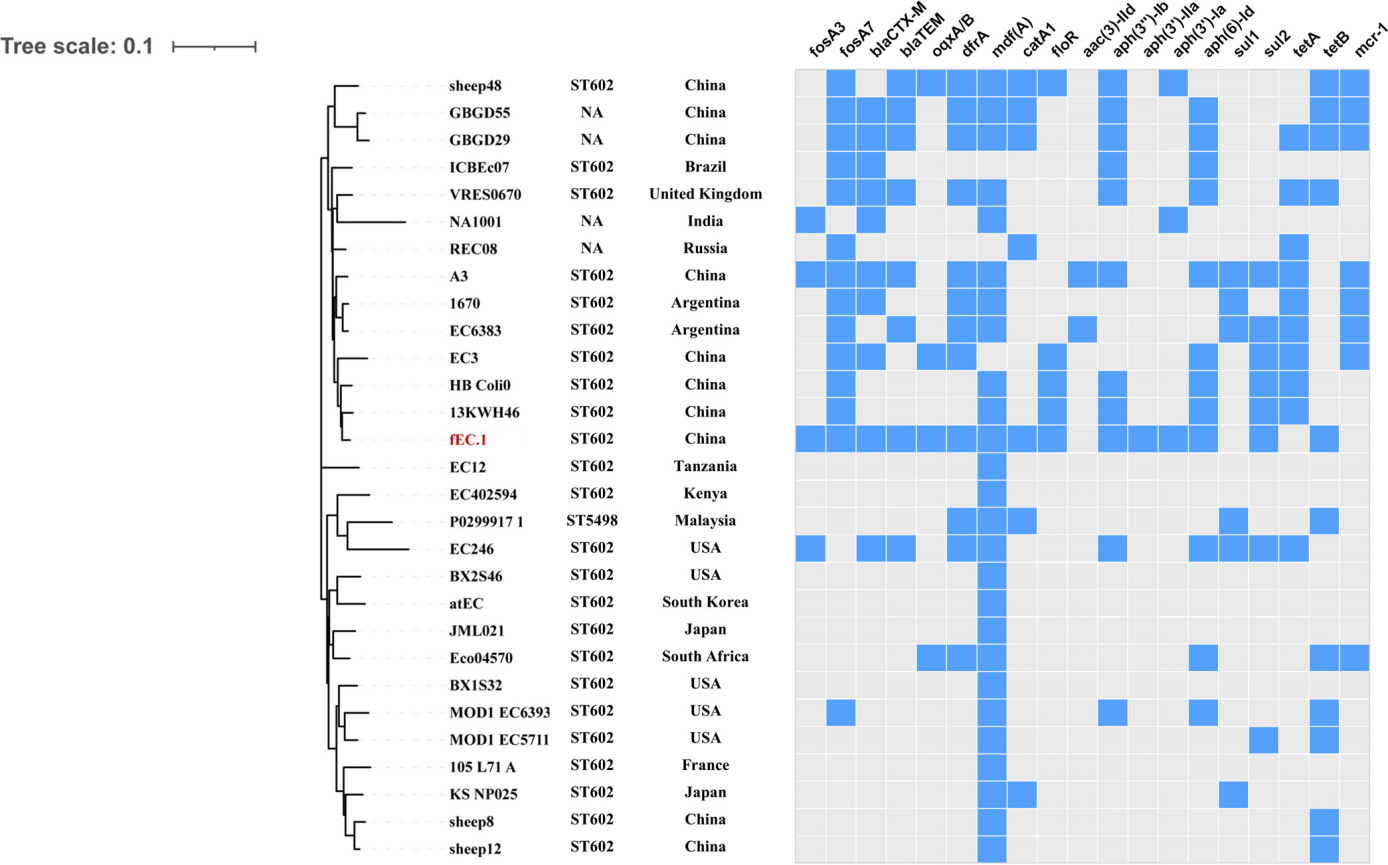
Phylogenetic relationship of ST602 E. coli isolate fEC.1 (in red) from this study with ST602 isolates from China and other countries. Blue and gray squares indicate the presence and absence of antimicrobial resistance genes, respectively.

Phylogenetic analysis was performed by using WGS information available in the GenBank database for ST602 fEC.1 and 28 E. coli isolates (ST602, *n* = 23; ST5498, *n* = 1; ST unknown, *n* = 4) from clinical samples from different sources, including humans, animals, and plants, and one E. coli isolate of unknown origin. The phylogenetic analysis by core genome MLST (cgMLST) revealed that ST602, ST5498 and 4 unknown STs were classified into the same lineage, indicating clonal spread of these strains. Isolate fEC.1 from this study was most closely related to two ST602 E. coli isolates, 13KWH46 (CP019250) and HB_Coli0 (CP020933), collected from a patient and chicken feces, which both carried *fosA7*, *mdf(A)*, *floR*, *aph(3′)-IIb*, *aph(6′)-Id*, *sul2*, *tet*(A), and *tet(B)* genes. Importantly, these strains were also abundant in distribution, including some countries in Asia, Africa, North America, and Europe, suggesting that an ST602 E. coli isolate is spreading across host species and continents. In addition, the majority of the strains carried fosfomycin resistance genes, including *fosA3* and *fosA7*, and showed a multiresistance gene profile (Fig. 8).

### Genetic background of *fosA7.5* in E. coli isolates.

**FIG 9:**
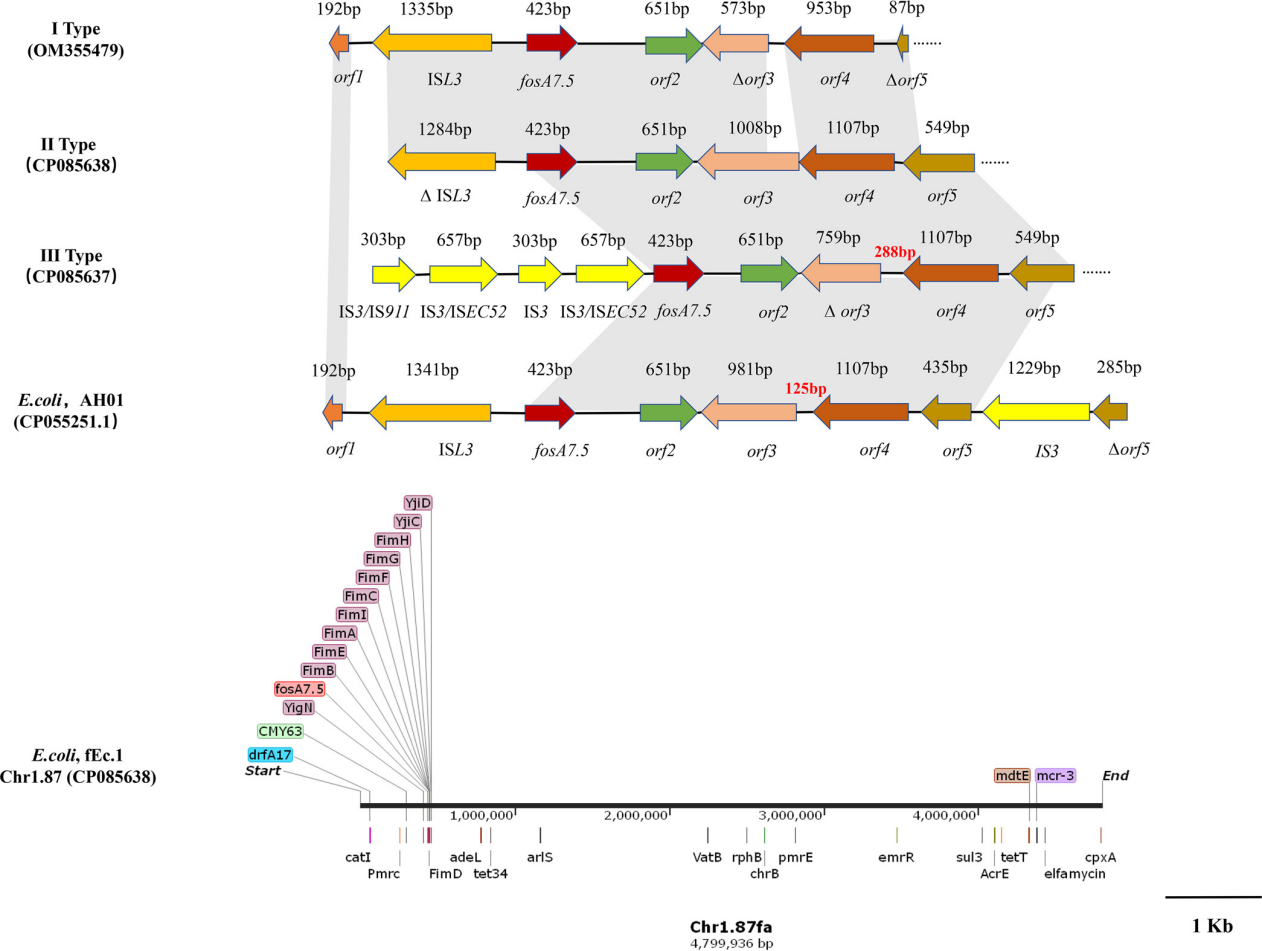
Genetic context of *fosA7.5* in E. coli. Arrows indicate the directions of transcription of the genes, and different genes are shown in different colors. Shaded boxes between sequences indicate homologous regions (>90% sequence identity). The letter Δ indicates a truncated gene.

In addition to strain fEC.1, a *fosA7.5*-carrying E. coli isolate (fEcg99-1) from a pigeon was also completely sequenced to analyze the genetic environment of *fosA7.5*. The MLST scheme revealed that fEcg99-1 belonged to sequence type ST2599. This study identified 3 different genetic contexts associated with the *fosA7.5* gene, designated type I to III (Fig. 9). WGS revealed that *fosA7.5* was located on the chromosome in strains fEc.1 (type II) and fEcg99-1 (type III). In all three types, a gene sequence containing *orf2*, *orf3*, *orf4*, and *orf5* was found downstream of *fosA7.5* that encoded HNH endonuclease, sialate-*O*-acetylesterase, sialic acid-induced transmembrane protein YjhT (NanM), and *N*-acetylneuraminic acid outer membrane channel protein (NanC), respectively. According to comparative genomic analysis, the three structures (type I to III) were highly similar to part of E. coli AH01 (CP055251.1). However, the difference was that the *orf3* sequence lengths in the three types were 573 bp, 1,008 bp, and 759 bp, respectively. The IS*L3* element was found in the upstream region of *fosA7.5* from isolates Kpg84, ECg85 (type I), and fEc.1 (type II), with lengths of 1,335 bp and 1,284 bp. Unlike the other two types, a sequence containing four IS*3*-like elements (IS*911*, 303 bp; IS*EC52*, 657 bp; IS911, 303 bp; and IS*EC52*, 657 bp) was confirmed upstream of *fosA7.5* in type III. Also, the mobile elements of type III were highly similar to the IS*3* element, located downstream of *fosA7.5* in E. coli AH01, but the genetic direction is opposite (Fig. 9).

The *fosA7.5* gene from E. coli in this study was 100% identical to *fosA7.5*^WT^ (wild-type *fosA7.5* sequence; WP_000941933.1), whereas it differed from the novel *fosA7* variant *fosA7.5*^Q86E^ (EC623772). The antimicrobial susceptibility testing also confirmed that the 10 *fosA7.5*-positive E. coli isolates in this study showed high-level resistance to fosfomycin (MIC ≥ 512 μg/mL). In addition, fimbriae proteins (FimB, FimA, and FimE), bacterial membrane proteins (YijC and YijN), along with T3SS and T6SS secretion systems were identified on *fosA7.5*-bearing chromosomes in strains fEC.1 and fECg99-1, all of which were connected to bacterial virulence. Moreover, genes for the two-component regulatory systems, resistance, and efflux pumps related to antibiotic resistance were also found on the chromosomes, for example, genes for the two-component regulatory system Arls, PmcR, and PmrE and the *mcr-3* gene involved in colistin resistance, as well as the ARGs *bla*_CMY-63_, *sul3*, *tetA*, and *dfrA* (Fig. 9).

### Phylogenetic analysis of fEC.99-1.

**FIG 10:**
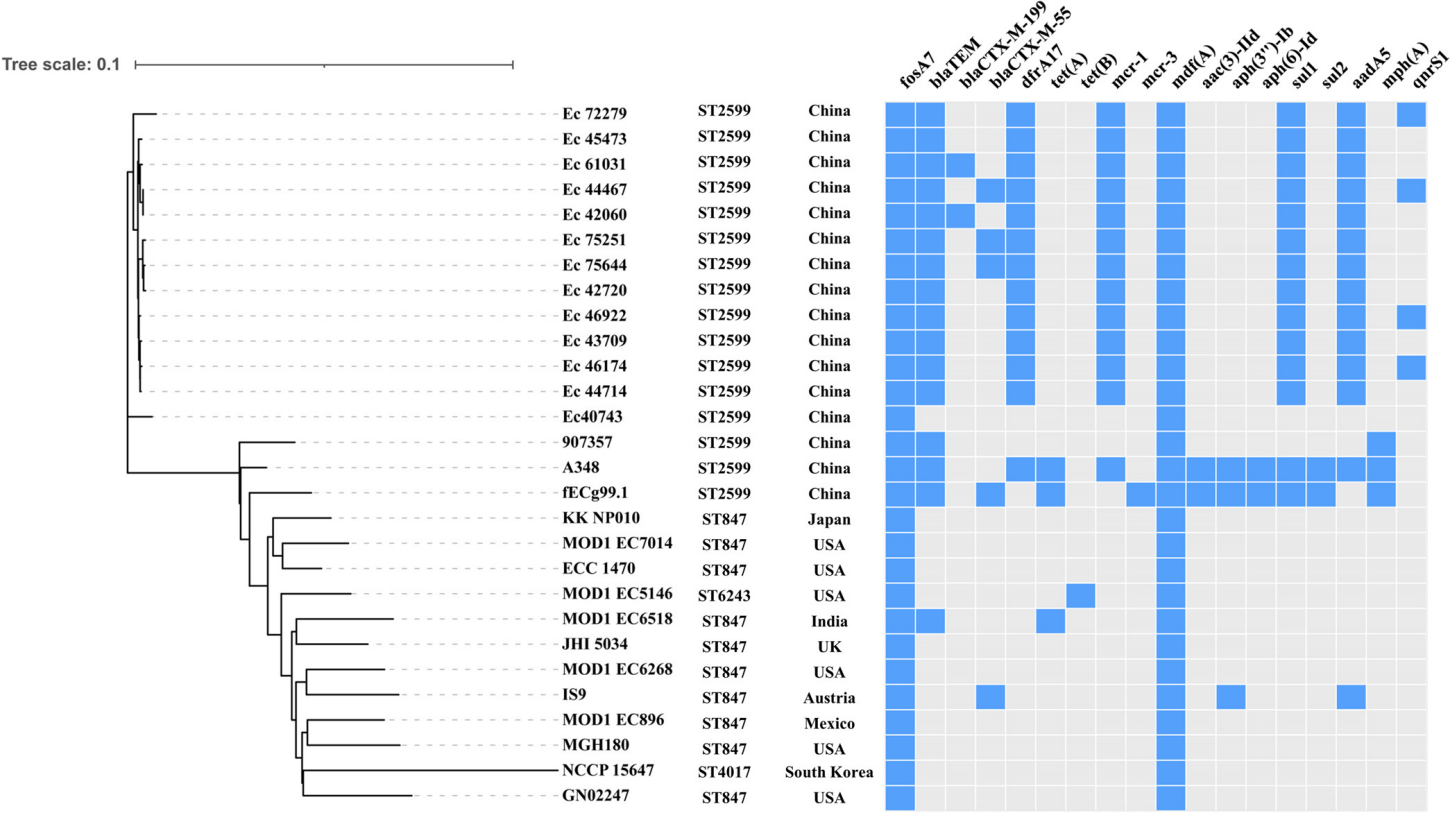
Phylogenetic relationship of ST2599 E. coli isolate fECg99-1 from this study with isolates from China and other countries. Blue and gray squares indicate the presence and absence of antimicrobial resistance genes, respectively.

Similarly, the *fosA7.5*-carrying E. coli ST2599 strain fECg99-1 was studied by core genome MLST (cgMLST)-based phylogenomic analysis with 27 E. coli strains in GenBank (ST2599, *n* = 15; ST847, *n* = 10; ST6243, *n* = 1; ST4017, *n* = 1). ST2599 and ST847 have 6 identical alleles and differ only in their *adk* alleles. The results showed that the E. coli isolates from different parts of the world and multiple sources (human, cow, chicken, mouse, and pigeon) clustered together. Isolate fECg99-1 was found to be in the same lineage as ST2599 isolates from humans, in which two isolates 907357 (AXUH01) and A348 (NSAT01) collected from China were most similar to fECg99-1, indicating that the E. coli ST2599 strain has spread between humans and animals. Moreover, ST847 E. coli from Australia, the United States, India, and Mexico shared clonal similarities with fEC.99-1. In addition, all strains carried *fosA7* and also showed a multiresistance gene profile (Fig. 10).

Finally, to determine whether *fosA7.5* in this study could confer resistance to fosfomycin, we created a recombinant plasmid, pET-28a+*fosA7.5* (Fig. S5). The fosfomycin MIC for E. coli Top10 transformed with pET-28a+*fosA7.5* was >128 μg/mL, which was more than 64-fold higher than that for E. coli Top10 transformed with pET-28a alone (Table 6).

**TABLE 6:** MICs for constructed and original strains

Antibiotic	MIC (μg/mL) for fEC.1		
	Alone	With pET-28a(+)-*fosA7.5*-Top10	With pET-28a(+)-Top10
Ceftazidime	32	<1	<1
Florfenicol	512	4	2
Chloramphenicol	256	2	2
Tetracycline	32	8	4
Ciprofloxacin	128	<1	<1
Amikacin	<1	<1	<1
Colistin	<1	<1	<1
Tigecycline	<0.25	<0.25	<0.25
Meropenem	<1	<1	<1
Ampicillin	>512	256	32
Fosfomycin	>512	>128	2

## DISCUSSION

Fosfomycin has been used all over the world to treat clinical urinary tract infections. However, with the irregular use of antibiotics, the problem of fosfomycin resistance has gradually become serious. The use of fosfomycin in veterinary medicine has not been approved in China. However, this study revealed that the *fosA*-like genes in animal-derived *Enterobacteriaceae* isolates have a general prevalence, with *fosA3* (26%) having the highest detection rate. This rate was higher than the previously reported positivity rate for *fosA3* in humans, ducks, and pets (7, 10, 19). In addition, all strains containing *fosA*-like genes in this study exhibited a high-level resistance to fosfomycin (MIC ≥ 512 μg/mL).

According to previous reports (20, 21), *fosA* was frequently discovered on the chromosome of E. cloacae or on the transposon Tn*2921* of S. marcescens, while data on *fosA* prevalence are lacking. In this study, a total of 20 *fosA*-positive strains were identified, with a rate of 10%. A recent study reported the discovery of *fosA* in pet-derived E. cloacae from Taiwan, China, and similar to this study, 2 strains carried both *fosA* and *fosA3* (18). In addition, 10 *fosA7.5*-positive E. coli isolates were obtained from pigeons, and three of them also harbored *fosA3*. Since the identification of *fosA7* on the chromosomes of Salmonella enterica serovar Heidelberg from chickens in 2017, it has been detected in different sources, such as humans and birds (17, 22). In a previous study (23), it was found that all *fosA7*-positive Salmonella isolates were susceptible to fosfomycin, whereas *fosA7.5* detected in this study can confer high-level fosfomycin resistance (MIC ≥ 512 μg/mL) in E. coli. It is worth noting that as avians, pigeons can transmit the strains carrying the *fosA7*-like gene into other natural habitats, which seems to provide a pathway for the spread of resistance genes. The above analysis shows that *fosA7* gene has appeared in food animals, birds, and environments where humans live, and pigeons might be considered a source or vector of resistant isolates posing a threat to public and animal health.

In this study, except for one strain that was resistant to only two antibiotics, all *fosA*/*fosA3*/*fosA7.5*-bearing *Enterobacteriaceae* isolates were MDR and displayed a high rate of resistance to ceftazidime, florfenicol, tetracycline, and ciprofloxacin. The high prevalence of drug resistance in fosfomycin-resistant strains may be related to the overuse of these drugs in farms. Furthermore, we also found that *fosA3* or *fosA7.5* was often coharbored with *bla*_CTX-M_, *floR*, and *bla*_TEM_ in the same strain, similar to a previous report (24), which is likely to facilitate the dissemination and maintenance of *fosA3* by coselection. However, the current study identified only 9 *rmtB*-producing isolates (9/52), which was in contrast to a prior study (25). In China, because of the widespread use of tetracycline, cephalosporins, aminoglycosides, and florfenicol as treatments or feed additives in animal husbandry, strains containing *fosA*-like genes have a high occurrence of other resistance genes (26). Therefore, limiting the use of antibiotics in animal agriculture may help prevent the spread of *fosA*-like genes in strains.

In this study, ERIC-PCR typing showed 6 unique clusters and 11 ERIC types for 52 *fosA3*-carrying E. coli isolates, which revealed genetic diversity. Moreover, some isolates had identical ERIC profiles, indicating dissemination from a similar origin. This result was consistent with a previous report that there was both clonal and horizontal transmission of these *fosA3*-positive E. coli (27). MLST analysis of 29 conjugable *fosA3*-positive E. coli isolates identified 15 STs, and ST115 was the most prevalent type, followed by ST156. However, ST115 and ST156 were previously found in ESBL-producing E. coli strains recovered from food and human samples (28, 29). MLST combined with ERIC-PCR analyses indicated that the 10 *fosA7.5*-positive E. coli isolates were mainly cloned among pigeons, which should arouse attention. At the same time, it demonstrated that the prevalence of fosfomycin-resistant strains has gradually increased, resulting in more serious problems of drug resistance.

The *fosA* gene was reported on conjugative plasmids or transposon Tn*2921* of S. marcescens strains (20, 21) in which the encoded protein FosA^Tn2921^ is closely related to FosA, encoded on the chromosome of E. cloacae, indicating that *fosA* has been transferred between strains. All *fosA*-positive isolates in this study showed high levels of resistance to fosfomycin, but no *fosA*-carrying transconjugants were obtained, implying that *fosA* might be located on the chromosomes or nonconjugative plasmids of these *Enterobacteriaceae* isolates. Upon analysis of the genetic environment of *fosA*, a partial sequence similar to the transposon Tn*2921* and E. cloacae ECNIH5 was found, which suggested that mobile elements or transposons were the primary reason for the extensive spread of *fosA* among *Enterobacteriaceae*.

Our findings showed that *fosA3* was successfully transferred from donors to the recipient E. coli C600, implying that *fosA3* could be horizontally transferred to different bacterial individuals. Furthermore, this work identified six genetic environments of *fosA3*, and *fosA3* was frequently flanked by IS*26*, consistent with previous studies (30). Besides IS*26*, the different mobile elements identified in the regions surrounding *fosA3* and other resistance genes by WGS analysis include IS*91*, IS*4*, IS*Vsa3*, and IS*1*. These elements might play an important role in spreading antimicrobial resistance genes in Gram-negative bacteria (31). In short, the diversity of genetic contexts reflects the complexity of *fosA3* transmission in E. coli. According to previous reports, the *fosA3*-carrying plasmids were mainly IncFII, IncN, and IncFIB plasmids (32). In this study, *fosA3* was discovered on the conjugative IncFII plasmid. Additionally, the full sequence comparison analysis of plasmid showed that the IncFII plasmid in this study has high homology (>99%) with other IncFII plasmids carrying *fosA3* from different sources, especially humans and chickens, suggesting that *fosA3*-bearing IncFII plasmids are widely present in animals and humans.

Contrary to previous reports (17), no *fosA7.5*-carryig transconjugants were obtained in this study. The *fosA7.5* gene was located on the chromosomes of E. coli isolates belonging to ST602 and ST2599 and shared 100% similarity with *fosA7.5*^WT^. This study showed that *fosA7.5* could confer resistance to fosfomycin, because of the amino acid difference between FosA7.5 found in E. coli and FosA7 first found in Salmonella serovar Heidelberg, which is a crucial factor for the *fosA7.5* gene to show resistance to fosfomycin in E. coli. In this study, the isolates frequently contained insertion sequences (IS*L3* and IS*3*) both upstream and downstream of *fosA7.5*. As previously reported (33), *fosA7* alleles on the chromosomes could act as reservoirs of potential resistance genes, and they can be captured by mobile genetic elements to horizontally disseminate between different bacteria. In addition, *fosA7.5*-positive E. coli ST602 and ST2599 were found to be clonally transmitted, leading to an increased risk of drug resistance transmission to humans via the food chain, which could pose a serious threat to public health.

In conclusion, this study revealed a high prevalence and complex genetic environment of *fosA*-like genes in farm samples. Whether *fosA*-like genes are located on the chromosomes or plasmids of isolates, they may spread, mediated by mobile elements. The fosfomycin resistance gene is potentially transferred to the human body through the food chain, thus increasing the risk for human public health, and should be regularly monitored.

## MATERIALS AND METHODS

### Bacterial strains.

From September 2019 to December 2020, a total of 531 samples were collected from animals (chicken, pig, and pigeon) and their surroundings (sewage and soil) in farms in Guangxi Province, China. All samples were screened for the presence of fosfomycin-resistant isolates. Briefly, the samples were placed into LB broth and shaken at 37°C for approximately 16 to 18 h. Then, the fosfomycin-resistant isolates were selected on xylose-lysine-deoxycholate (XLD) agar plates (*Enterobacteriaceae* identification medium) containing 256 μg/mL fosfomycin. From each sample, only a single isolate of any one species was obtained. The strains were further identified using 16S rRNA sequencing (34), using primers described previously (F, AGAGTTTGATCATGGCTC; R, GGTTACCTTGTTACGACTT).

### Identification of fosfomycin-resistant determinants and the coexisting resistance genes.

The existence of fosfomycin-modifying-enzyme genes (*fosA3*, *fosA*, *fosC2*, *fosA7.5*, and others) in all selected fosfomycin-resistant isolates was determined by PCR and sequencing (18), and the *fosA7.5* primer was designed based on the sequence of *fosA7* (17). The surrounding regions of the *fosA*-like genes were determined by PCR mapping and sequencing using previously published primers (18, 24). Furthermore, the florfenicol resistance gene *floR*, the 16S rRNA methyltransferase gene *rmtB*, the carbapenem resistance gene *bla*_NDM_, the ESBL genes *bla*_CTX-M_ (groups 1, 2, 8, and 9) and *bla*_TEM_, and the plasmid-mediated AmpC lactamase gene *bla*_CMY-2_ were also identified using PCR and sequencing (35–38). All primers are listed in the supplemental material.

### Antimicrobial susceptibility testing.

The MICs of 12 antibiotics (ceftazidime, florfenicol, chloramphenicol, erythromycin, tetracycline, ciprofloxacin, amikacin, meropenem, colistin, tigecycline, fosfomycin, and rifampicin) for the *fosA*-like gene-positive isolates were determined by the agar dilution method or broth microdilution method according to the CLSI (39). The MICs of fosfomycin were determined by the agar dilution method on Mueller-Hinton agar supplemented with 25 μg/mL glucose-6-phosphate (G-6-P), and the resistant breakpoints were recommended by the EUCAST in 2020 (40). E. coli ATCC 25922 was used as the control strain.

### Conjugation assays and plasmid replicon typing.

The transferability of fosfomycin resistance genes was determined by broth mating method using the plasmid-free E. coli C600 strain (Rif^r^) as the recipient. Transconjugants were selected on MacConkey agar plates containing fosfomycin (100 μg/mL), G-6-P (25 μg/mL), and rifampicin (250 μg/mL) and finally confirmed by ERIC-PCR. When the conjugation experiments failed, E. coli DH5α was used as the recipient for transformation experiments. The transfer of the fosfomycin resistance genes (*fosA*, *fosA3*, or *fosA7.5*) was confirmed by PCR, and the MICs of transconjugants were also detected as described above. PCR-based replicon typing (PBRT) was used to screen the plasmid incompatibility groups for the *fosA*-like gene-positive isolates and their corresponding transconjugants (41). The primers are listed in the supplemental material.

### MLST and ERIC-PCR.

The 29 conjugable *fosA3*-positive E. coli isolates and the 10 *fosA7.5-*harboring isolates were subjected to MLST analysis, which was performed as previously described (42). The STs were obtained from the MLST database website (http://mlst.warwick.ac.uk/mlst/dbs/Ecoli). ERIC-PCR was carried out by using the primers ERIC-1 and ERIC-2 for *fosA3*-positive and *fosA7.5*-positive E. coli isolates (43). The isolated *Enterobacteriaceae* DNA samples were amplified in order to construct a computerized dendrogram, with the presence and absence of bands assumed to be 1 and 0, respectively. Following software processing, a matrix diagram of the binary number sequence was created and imported into NTSYS-pc (version 2.10) to perform the cluster analysis (44), which is based on the unweighted pair group method with arithmetic averages (UPGMA). Cluster were defined as being the same when the similarity between ERIC-PCR profiles was >80%.

### Whole-genome sequencing and phylogenetic analysis.

Whole-genome sequencing of two representative E. coli isolates (fEC.1 and fEC.99-1) from pigeons was performed. The extracted total genomic DNA of isolates was sequenced using the Nanopore PromethION and Illumina NovaSeq PE150 sequencing platforms, and the reads were assembled using Unicycler software. The coding sequences of the genetic context surrounding *fosA3* and *fosA7.5* were analyzed using the ORF Finder program (www.ncbi.nlm.nih.gov/gorf/orfig.cgi), and annotation was performed using the RAST server (http://rast.nmpdr.org/). The plasmid replicon types and antibiotic resistance genes prediction were analyzed using tools found at http://pubmlst.org/plasmid/ and https://cge.cbs.dtu.dk/services/. Genome comparison analysis of plasmids was performed using Easyfig and BRIG. WGS information for E. coli isolates was downloaded from GenBank (Tables S5 and S6), and cgMLST was performed as described previously (45).

### Cloning, expression, and functional verification of *fosA7.5*.

The *fosA7.5* gene from E. coli fEC.1 was cloned into pET-28a(+) and was transferred into E. coli Top10 by heat shock. Transformants were selected on LB agar plates containing 100 μg/mL kanamycin. Then, the recombinant clones were identified by PCR and Sanger sequencing. The Top10 strain containing pET-28a(+)-*fosA7.5* and the Top10 control strain were subjected to a fosfomycin resistance test to verify it’s functionality.

### Data availability.

The *fosA7.5*-bearing chromosome sequences of fEc.1 and fEcg99-1 were submitted to NCBI with the accession numbers CP085638 and CP085637, respectively. The *fosA3*-bearing plasmid (pfEc.1-3) sequence was submitted with the accession number OK605583. The nucleotide sequences of *fosA* (types I, II, III, and IV), *fosA3* (types I, II, IV, V, and VI), and *fosA7.5* (type I) in this study have been deposited in GenBank under the accession numbers OM355477, OM289150, OM289151, OM355478, OM420281, OM355482, OM355480, OM355481, OM355483, and OM355479.

## Supplementary Material

Reviewer comments
