# Supplementary material for: Spread and Molecular Characteristics of Enterobacteriaceae Carrying fosA-Like Genes from Farms in China
Source: Microbiol Spectr. 2022 Jul 19;10(4):e00545-22. doi: 10.1128/spectrum.00545-22 (PMC9431306; doi:10.1128/spectrum.00545-22)
Supplement: Supplemental file 1 — Supplemental material. Download spectrum.00545-22-s0001.pdf, PDF file, 0.7 MB [file spectrum.00545-22-s0001.pdf]

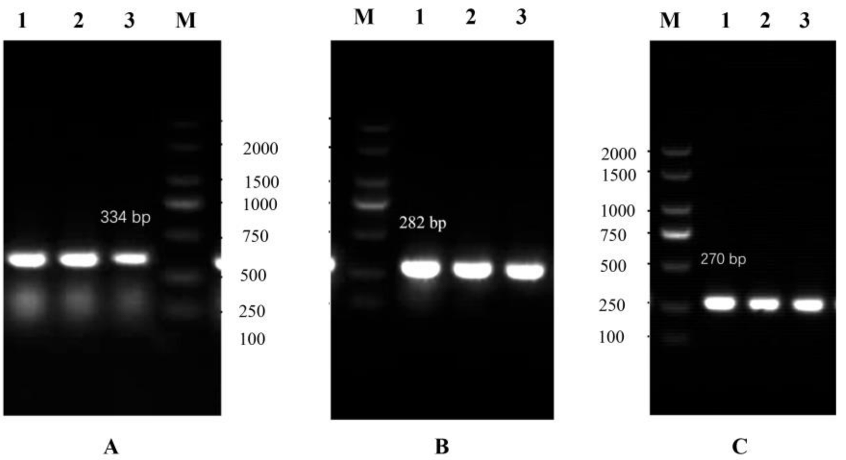

Figure S1 Amplification of *fosA* (A)、*fosA3* (B) and *fosA7.5* (C) genes of some strains
Note: M: DL 2000 DNA Marker; 1-3 Lanes: fosA like genes-positive strains

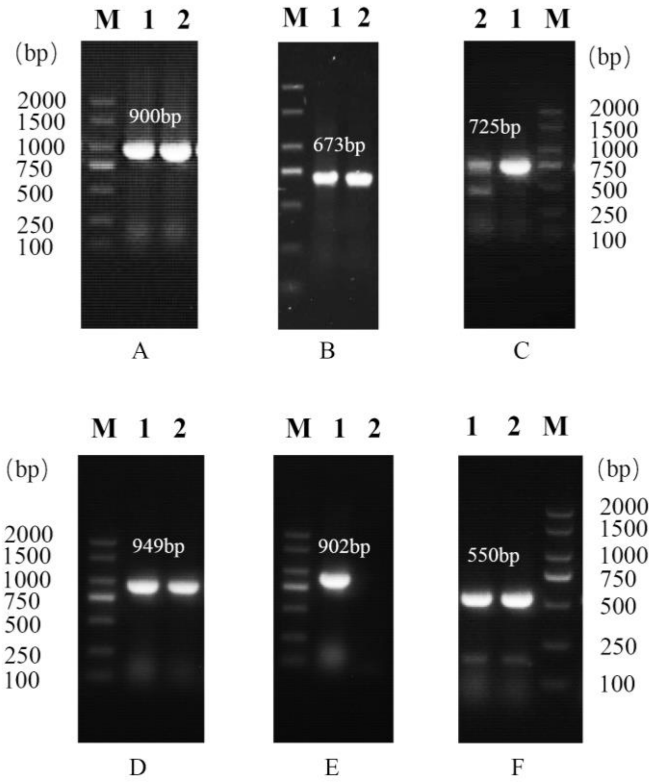

Figure S2 Electrophoresis results of *bla*$_{\text{NDM}}$ (A), *floR* (B), *rmtB* (C), *bla*$_{\text{CTX-M-1}}$ (D), *bla*$_{\text{CTX-M-9}}$ (E) and *bla*$_{\text{TEM}}$ (F) genes of strains
Note: M: DL 2000 DNA Marker; 1-2 Lanes: fosfomycin-resistant strains

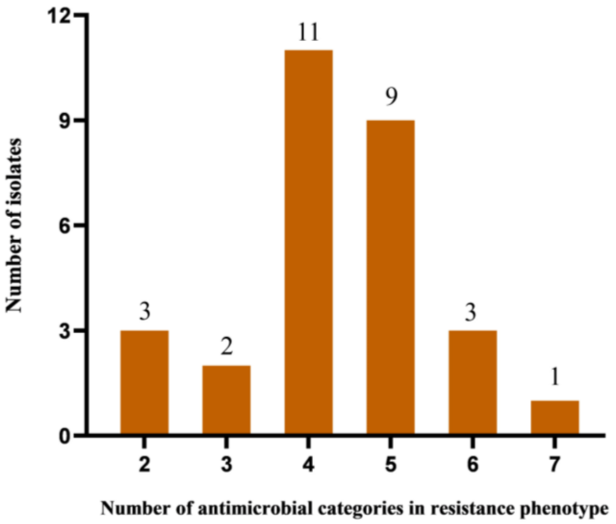

Figure S3 The number of strains of 29 conjugations in different resistant type

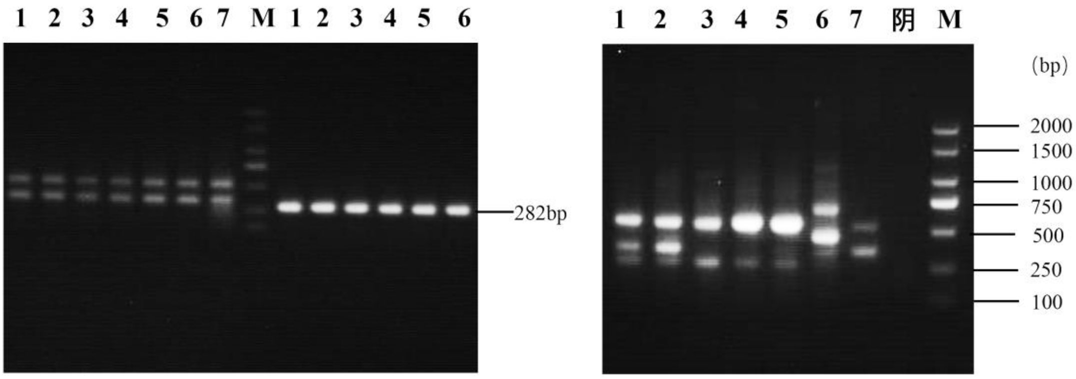

Figure S4 Electrophoresis results of C600, conjugations ERIC-PCR and *fosA3* of conjugations (left); ERIC-PCR electrophoresis results of donor isolates and C600 (right)

Note: M: DL 2000 DNA Marker; 1-7: E27-T, E28-T, E29-T, E30-T, E31-T, fEC.1-T, C600

Table S1 Information of 82 strains carrying fosfomycin resistance genes

Strains	Species	sources	*fosA*-like gene	Strains	Species	sources	*fosA*-like gene
EC1	*E. coli*	pigeon	*fosA3*	EC42	*E. coli*	chicken, feces	*fosA3*
EC2	*E. coli*	chicken, feces	*fosA3*	EC43	*E. coli*	chicken, feces	*fosA3*
EC3	*E. coli*	chicken, feces	*fosA3*	EC44	*E. coli*	chicken, feces	*fosA3*
EC4	*E. coli*	chicken, feces	*fosA3*	EC45	*E. coli*	chicken, feces	*fosA3*
EC5	*E. coli*	chicken, feces	*fosA3*	EC46	*E. coli*	chicken, feces	*fosA3*
EC6	*E. coli*	chicken, feces	*fosA3*	EC47	*E. coli*	chicken, feces	*fosA3*
EC7	*E. coli*	pig, sewage	*fosA3*	EC48	*E. coli*	chicken, feces	*fosA3*
EC8	*E. coli*	chicken, feces	*fosA3*	EC49	*E. coli*	pig, nose	*fosA3*
EC9	*E. coli*	chicken, feces	*fosA3*	EC50	*E. coli*	chicken, mouth	*fosA3*
EC10	*E. coli*	chicken, feces	*fosA3*	EC51	*E. coli*	chicken, feces	*fosA3*
EC11	*E. coli*	chicken, feces	*fosA3*	EC52	*E. coli*	chicken, feces	*fosA3*
EC12	*E. coli*	chicken, feces	*fosA3*	20E. 1	*E. hormaechei*	pig, soil	*fosA*
EC13	*E. coli*	chicken, feces	*fosA3*	20E. 2	*E. cloacae*	chicken, soil	*fosA*
EC14	*E. coli*	pig, feces	*fosA3*	EC2088	*E. hormaechei*	chicken, feces	*fosA*
EC15	*E. coli*	chicken, feces	*fosA3*	20E. 4	*E. hormaechei*	chicken, feces	*fosA*
EC16	*E. coli*	chicken, feces	*fosA3*	20E. 5	*E. hormaechei*	chicken, soil	*fosA*
EC17	*E. coli*	pig, lungs	*fosA3*	20E. 6	*E. cloacae*	pig, sewage	*fosA*
EC18	*E. coli*	pigeon	*fosA3*	20E. 7	*E. asburiae*	chicken, soil	*fosA*
EC19	*E. coli*	pig, mouth	*fosA3*	20E. 8	*E. asburiae*	chicken, soil	*fosA*
EC20	*E. coli*	pig, lungs	*fosA3*	20E. 9	*E. asburiae*	chicken, feces	*fosA*
EC21	*E. coli*	pig, feces	*fosA3*	EC2098	*E. hormaechei*	chicken, feces	*fosA*
EC22	*E. coli*	chicken, feces	*fosA3*	20E. 11	*E. cloacae*	pig, soil	*fosA*
EC23	*E. coli*	pig, mouth	*fosA3*	KP20117	*E. cloacae*	pig, soil	*fosA*
EC24	*E. coli*	pigeon	*fosA3*	20E. 13	*E. cloacae*	pig, mouth	*fosA*
EC25	*E. coli*	chicken, feces	*fosA3*	20E. 14	*E. cloacae*	pig, feces	*fosA*
EC26	*E. coli*	pig, mouth	*fosA3*	20E. 15	*E. cloacae*	pig, lungs	*fosA*, *fosA3*
EC27	*E. coli*	chicken, feces	*fosA3*	20E. 16	*E. cloacae*	egg shell	*fosA*, *fosA3*
EC28	*E. coli*	pig, sewage	*fosA3*	20E. 17	*E. cloacae*	chicken, soil	*fosA*
EC29	*E. coli*	chicken, feces	*fosA3*	20E. 18	*E. hormaechei*	pig, nose	*fosA*
EC30	*E. coli*	pig, anus	*fosA3*	EC1928	*E. hormaechei*	pig, feces	*fosA*
EC31	*E. coli*	pig, nose	*fosA3*	20E. 20	*E. cloacae*	chicken, soil	*fosA*
EC32	*E. coli*	chicken, feces	*fosA3*	Kpg84	*E. coli*	pigeon	*fosA3*, *fosA7.5*
EC33	*E. coli*	pig, soli	*fosA3*	fEc. 1	*E. coli*	pigeon	*fosA3*, *fosA7.5*
EC34	*E. coli*	chicken, feces	*fosA3*	ECg85	*E. coli*	pigeon	*fosA3*, *fosA7.5*
EC35	*E. coli*	pig, mouth	*fosA3*	fEcg99-1	*E. coli*	pigeon	*fosA7.5*
EC36	*E. coli*	pig, lungs	*fosA3*	ECg29	*E. coli*	pigeon	*fosA7.5*
EC37	*E. coli*	pig, feces	*fosA3*	ECg931	*E. coli*	pigeon	*fosA7.5*
EC38	*E. coli*	chicken, feces	*fosA3*	ECg932	*E. coli*	pigeon	*fosA7.5*
EC39	*E. coli*	chicken, feces	*fosA3*	ECg91	*E. coli*	pigeon	*fosA7.5*
EC40	*E. coli*	chicken, feces	*fosA3*	ECg933	*E. coli*	pigeon	*fosA7.5*
EC41	*E. coli*	pig, nose	*fosA3*	EC315	*E. coli*	pigeon	*fosA7.5*

Table S2 MICs (μg/mL) of 12 antimicrobial agents for 52*fosA3*-carrying strains

Strains	CAZ	FFC	CHL	TET	CIP	AMK	MEM	COL	TGC	RIF	FOS
EC1	32	256	128	128	8	2	<1	<1	0.5	3.9	>512
EC2	16	32	16	512	512	4	<1	<1	1	31.2	>512
EC3	64	>512	>512	512	16	<1	<1	<1	2	500	>512
EC4	16	256	128	128	16	4	<1	<1	0.5	250	>512
EC5	8	>512	512	512	128	4	<1	<1	1	15.625	>512
EC6	16	>512	512	512	256	2	<1	<1	2	15.625	>512
EC7	<1	256	128	32	<1	<1	<1	<1	<0.25	7.8	>512
EC8	16	128	64	8	8	<1	<1	<1	0.5	125	>512
EC9	32	256	256	64	16	<1	<1	<1	<0.25	7.8	>512
EC10	<1	512	512	64	<1	<1	<1	<1	<0.25	7.8	>512
EC11	<1	512	256	128	2	<1	<1	<1	<0.25	7.8	>512
EC12	<1	32	128	16	64	<1	<1	>512	4	15.625	>512
EC13	16	256	128	16	16	4	<1	<1	0.5	125	>512
EC14	8	512	512	512	128	4	<1	<1	1	31.25	>512
EC15	8	>512	512	512	256	2	<1	<1	1	31.25	>512
EC16	<1	128	64	32	4	2	<1	<1	<0.25	7.8	>512
EC17	128	>512	512	64	128	>512	<1	<1	2	7.8	>512
EC18	16	512	512	32	32	4	<1	<1	<0.25	3.9	>512
EC19	<1	>512	256	16	16	<1	<1	<1	0.5	15.625	>512
EC20	128	>512	512	128	256	>512	<1	<1	0.5	7.8	>512
EC21	32	512	256	256	128	2	<1	<1	<0.25	15.625	>512
EC22	128	>512	128	256	256	>512	<1	<1	1	7.8	>512
EC23	128	>512	512	128	256	>512	<1	<1	1	7.8	>512
EC24	<1	16	128	16	32	<1	<1	>512	8	15.625	>512
EC25	<1	128	64	32	8	<1	<1	<1	<0.25	3.9	>512
EC26	128	>512	512	128	128	>512	<1	<1	2	7.8	>512
EC27	<1	256	64	64	16	<1	<1	0.5	<0.25	7.8	512
EC28	2	256	64	128	8	2	<1	1	<0.25	3.9	>512
EC29	32	256	64	64	8	<1	<1	<1	1	7.8	512
EC30	32	512	128	256	<1	2	<1	2	<0.25	7.8	512
EC31	32	512	128	128	<1	2	<1	0.5	<0.25	7.8	>512
EC32	32	256	64	64	16	8	<1	0.5	2	3.9	>512
EC33	64	256	128	64	32	2	<1	0.5	<0.25	7.8	>512
EC34	<1	256	64	128	4	<1	<1	<1	1	3.9	>512
EC35	<1	>512	128	256	16	<1	<1	<1	1	15.625	>512
EC36	32	>512	256	128	64	>512	<1	1	<0.25	7.8	>512
EC37	64	>512	512	512	64	2	<1	1	<0.25	7.8	>512
EC38	<1	512	256	256	2	2	<1	0.5	<0.25	7.8	512
EC39	16	512	128	512	128	<1	<1	<1	2	7.8	>512
EC40	16	>512	256	512	256	<1	<1	1	<0.25	7.8	>512
EC41	32	>512	256	512	32	<1	<1	<0.25	2	3.9	>512
EC42	<1	256	64	64	16	<1	<1	<1	<0.25	7.8	>512

EC43	16	128	64	64	32	4	<1	2	1	7.8	>512
EC44	16	512	128	64	8	4	<1	0.5	<0.25	7.8	>512
EC45	32	512	64	128	64	2	<1	<0.25	<0.25	3.9	>512
EC46	16	>512	256	512	256	2	<1	2	<0.25	15.625	>512
EC47	8	512	128	64	32	<1	<1	<1	1	3.9	>512
EC48	16	512	128	128	64	4	<1	1	<0.25	3.9	>512
EC49	32	>512	256	512	16	<1	<1	<0.25	<1	3.9	>512
EC50	<1	256	128	128	32	<1	<1	<0.25	0.5	7.8	>512
EC51	<1	256	64	128	4	<1	<1	<1	2	3.9	>512
EC52	8	>512	256	256	2	>512	<1	<1	2	7.8	512

Tabl3 S3 MICs (μg/mL) of 12 agents for 20 *fosA*-carryings and 10 *fosA7.5*-carrying strains

Strains	CAZ	FFC	CHL	TET	CIP	AMK	MEM	COL	TGC	RIF	FOS
20E.1	<1	8	8	8	<1	<1	<1	<1	1	15.625	>512
20E.2	<1	8	4	8	<1	<1	<1	<1	2	15.625	>512
EC2088	<1	512	128	128	<1	<1	<1	<1	4	>1000	>512
20E.4	<1	512	128	256	<1	<1	<1	<1	4	1000	>512
20E.5	<1	512	128	256	<1	<1	<1	<1	4	1000	>512
20E.6	<1	8	8	8	<1	<1	<1	64	2	15.625	>512
20E.7	<1	2	4	8	<1	<1	<1	64	2	15.625	>512
20E.8	<1	8	8	16	<1	<1	<1	32	2	15.625	>512
20E.9	2	>512	256	256	4	<1	<1	<1	0.5	>512	>512
EC2098	<1	512	128	64	<1	<1	<1	<1	1	7.8	512
20E.11	2	512	128	128	<1	<1	<1	2	2	31.25	>512
KP20117	16	512	256	512	8	2	<1	64	2	500	>512
20E.13	32	512	512	512	8	<1	<1	64	16	500	>512
20E.14	256	>512	>512	>512	>512	>512	<1	<1	0.5	500	>512
20E.15	<1	32	128	16	64	<1	<1	>512	4	15.625	>512
20E.16	128	512	128	512	<1	<1	<1	64	8	>1000	>512
20E.17	>512	<1	<1	128	<1	<1	8	<1	0.5	7.8	>512
20E.18	<1	512	256	256	4	4	<1	<1	2	500	>512
EC1928	<1	8	2	8	<1	<1	<1	16	2	15.625	>512
20E.20	32	512	512	32	128	2	<1	<1	<0.25	3.9	>512
Kpg84	32	512	256	32	128	<1	<1	<1	<0.25	3.9	>512
fEc. 1	64	512	256	128	64	2	<1	<1	<0.25	3.9	>512
ECg85	32	256	256	128	64	2	<1	<1	<0.25	3.9	>512
fEcg99-1	16	64	64	128	8	4	<1	<1	<0.25	<1.95	>512
ECg29	32	512	128	64	8	4	<1	<1	<0.25	3.9	>512
ECg931	32	512	128	128	8	4	<1	<1	<0.25	3.9	>512
ECg932	16	64	256	64	8	16	1	<1	<0.25	<1.95	>512
ECg91	32	256	128	128	8	2	<1	<1	0.5	3.9	>512
ECg933	<1	256	8	4	<1	<1	<1	<1	<0.25	<1.95	512

Note: CAZ, Ceftazidime; FFC, Florfenicol; CHL, chloramphenicol; MXF, erythromycin; TET, tetracycline; CIP, ciprofloxacin; AMK, amikacin; MEM, meropenem; COL, colistin; TCG, tigecycline; RIF, rifampicin; FOS, fosfomycin.

Table S4 Plasmid replicons of the 10 *fosA7.5*-Positive *E. coli* and their transconjugants

Strains	Plasmid type	transconjugants	Plasmid type
Kpg84	FrepB，FIB，FII，I1，K	Kpg84-T	I1，FIB，FII
fEc.1	FrepB，FIB，FII，I1，K	Ecg87-T	I1，FIB，FII
ECg85	FrepB，FIB，I1，FII，K	Kpg85-T	I1，FIB，FII
fEcg99-1	FrepB，FIB，I1，Y，FII，K	None	None
ECg29	FrepB，FIB，I1，Y，FII，K	None	None
ECg931	FrepB，FIB，I1，Y，FII，K	None	None
ECg932	FrepB，FIB，I1，Y，FII，K	None	None
ECg91	FrepB，FIB，I1，Y，FII，K	None	None
ECg933	FrepB，FIB，I1，Y，FII，K	None	None
EC315	FrepB，FIB，FII，K	None	None

Table S5 Information for 28 *E. coli* isolates in GenBank database

Strain	Accession number	Host	Species	Create Date	Isolation Source
sheep48	LVQR01	Sheep	*E. coli*	2013	feces
GBGD55	RQUV01	Human	*E. coli*	2014	Gut
GBGD29	RQTV01	Human	*E. coli*	2015	Gut
ICBEc07	NIHJ01	Human	*E. coli*	2017	Rectal swab
VRES0670	UDII01	Chicken	*E. coli*	2015	meat
NA1001	MKWV01	Chicken	*E. coli*	2015	Large intestine
RCE08	LAXB01	Human	*E. coli*	2013	Gut
A3	NSAG01	Human	*E. coli*	2016	feces
1670	PIIZ01	Human	*E. coli*	2016	urine
6383	PIJR01	Human	*E. coli*	2014	urine
EC3	QXGO01	Chicken	*E. coli*	2014	retailed food
HB_Coli0	CP020933	Chicken	*E. coli*	2017	Gut
13KWH46	CP019250	Human	*E. coli*	2013	urine
fEC.1	CP085638	Pigeon	*E. coli*	2020	Organs
EC12	LIQA01	Cow	*E. coli*	2015	Unknown
402594	LGMW01	Human	*E. coli*	2007	feces
P0299917_1	AQER01	Human	*E. coli*	2011	feces
EC246	SBKI01	Unknown	*E. coli*	2019	Unknown
BX2S46	SRNQ01	Plant	*E. coli*	2019	retail fresh oregano
atEC	LRBX01	Mouse	*E. coli*	2014	feces

JML021	BFMS01	Human	*E. coli*	2015	Unknown
Eco04570	CACRYI01	Human	*E. coli*	2016	feces
BX1S32	SRND01	Plant	*E. coli*	2019	retail fresh mint
MOD1_EC6393	NLYN01	Pig	*E. coli*	2017	Lung
MOD1_EC5711	NLHB01	Cow	*E. coli*	2019	feces
105_L71_A	BIBS01	Cow	*E. coli*	2011	Unknown
KSNP025	BFYM01	Cow	*E. coli*	2013	Unknown
sheep8	LVPD01	Sheep	*E. coli*	2013	feces
sheep12	LVPH01	Sheep	E. coli	2013	feces

Table S6 Information for 27 *E. coli* isolates in GenBank database

Strain	Accession number	Host	Species	Create Date	Isolation Source
Ec_72279	VLOL01	Human	*E. coli*	2018	urine
Ec_45473	VLOR01	Human	*E. coli*	2017	urine
Ec_61031	VLPH01	Human	*E. coli*	2016	stool
Ec_44467	VLOU01	Human	*E. coli*	2017	urine
Ec_42060	VLPD01	Human	*E. coli*	2016	urine
Ec_75251	VLOJ01	Human	*E. coli*	2018	urine
Ec_75644	VLOI01	Human	*E. coli*	2018	urine
Ec_42720	VLPA01	Human	*E. coli*	2016	sputum
Ec_46922	VLON01	Human	*E. coli*	2017	urine
Ec_43709	VLOX01	Human	*E. coli*	2016	sputum
Ec_46174	VLOQ01	Human	*E. coli*	2017	urine
Ec_44714	VLOT01	Human	*E. coli*	2017	sputum
Ec40743	CP041919	Human	*E. coli*	2016	sputum
907357	AXUH01	Human	*E. coli*	/	/
A348	NSAT01	Human	*E. coli*	2016	feces
fECg99.1	CP085637	Pigeon	*E. coli*	2020	Organs
KK_NP010	BFWB01	Cow	*E. coli*	2014	/
MOD1_EC7014	NMMX01	Cow	*E. coli*	/	milk
ECC_1470	CP010344	Cow	*E. coli*	/	udder
MOD1_EC5146	NOGI01	Oreotragus	*E. coli*	2007	feces
MOD1_EC6518	NLVX01	Chicken	*E. coli*	/	blood
JHI_5034	OEDN01	/	*E. coli*	2008	barley trial
MOD1_EC6268	NMAH01	Cow	*E. coli*	2011	small intestine
IS9	CBWC01	Human	*E. coli*	2013	/
MOD1_EC896	NJJO01	Mouse	*E. coli*	2002	feces
MGH180	NGSF01	Human	*E. coli*	2015	/
NCCP_15647	AJMB01	Human	*E. coli*	2003	feces
GN02247	LQSP01	Human	*E. coli*	2003	blood

The primer sequence and fosfomycin resistance genes

Primer name	Sequence (5'-3')	product size (bp)	Tm (℃)
fosA-F	CTCAACCATCTGACCCTCGC	334	58
fosA-R	CGTGCAGCTCCAGCTTGT		
fosA2-F	GCTGCAATCACTCAACCATC	346	54
fosA2-R	CACGTGCAGCTCCAGCTT		
fosA3-F	GCGTCAAGCCTGGCATTT	282	57
fosA3-R	GCCGTCAGGGTCGAGAAA		
fosA4-F	CTGGCGTTTTATCAGCGGTT	230	55
fosA4-R	CTTCGCTGCGGTTGTCTTT		
fosA5-F	TATTAGCGAAGCCGATTTTGCT	187	55
fosA5-R	CCCCTTATACGGCTGCTCG		
fosA6-F	GCTACGGTTCAGCTTCCAGA	242	56
fosA6-R	CGAGCGTGGCGTTTTATCAG		
fosC2-F	TGGAGGCTACTTGGATTTG	217	50
fosC2-R	AGGCTACCGCTATGGATTT		
fosA8-F	ACGCCCTTAACCATCTGACCC	307	55
fosA8-R	AGAAAATAGAACGACGCCCCTT		
fosA10-F	CGAGCGTGGCGTTTTATCAG	278	53
fosA10-R	GCCATCGGGATCGAGGAAAT		
fosA7-F	CTCTGAACCACTTAACGCT	270	53
fosA7-R	CTTTCCAGACCGTCACTCC		
16S-27F	AGAGTTTGATCCTGGCTCAG	1500	58
16S-1492R	GGTTACCTTGTTACGACTT		

The primer sequence of oter resistance genes

Primer name	Sequence (5'-3')	product size (bp)	Tm (℃)
CTX-M-1-F	TTCCAGAATAAGGAATCCC	949	55
CTX-M-1-R	CGTCTAAGGCGATAAACAAA		
CTX-M-9-F	TGACCGTATTGGGAGTTTG	902	56
CTX-M-9-R	ACCAGTTACAGCCCTTCG		
CTX-M-2-F	CGACGCTACCCCTGCTATT	552	58
CTX-M-2-R	CCAGCGTCAGATTTTTCAGG		
TEM-F	AGGAAGAGTATGATTCAACA	550	58
TEM-R	CTCGTCGTTTGGTATGGC		
CMY2-F	AACAACTGATTGCGTCTGA	1228	62
CMY2-R	TCCTGGGCCTCATCGTCAGTTAT		
rmtB-F	ACATCAACGATGCCCTCAC	725	55
rmtB-R	AAGTTCTGTTCCGATGGTC		
floR-F	CTGAGGGTGTCGTCATCTAC	673	59
floR-R	GTCCCGACAATGCTGACTAT		
NDM-F	GAATTCGCCCCATATTTTTGC	900	56
NDM-R	AACGCCTCTGTCACATCGAAAT		

The primers for MLST of *Escherichia coli*

Primer name	Sequence (5'-3')	product size (bp)	Tm (°C)
adk-F	CTCGCCATTAACCGTTTCAG	736	55
adk-R	CCAGATCAGCGCGAACTTCA		
fumC-F	TCACAGGTCGCCAGCGCTTC	769	62
fumC-R	TCCCGGCAGATAAGCTGTGG		
gyrB-F	ATCGGCGACACGGATGAC	816	63
gyrB-R	GTCCATGTAGGCGTTCAGG		
icd-F	CCGGCACAAGGCAAGAAGATC	857	66
icd-R	GGACGCAGCAGGATCTGTT		
mdh-F	GCCTTCAGGTTCAGAACTCTCTCT	798	63
mdh-R	TTCTGTTCAAATGCGCTCAGG		
purA-F	CGCGCTGATGAAAGAGATGA	817	66
purA-R	CATACGGTAAGCCACGCAGA		
recA-F	CGCATTCGCTTTACCCTGACC	731	53
recA-R	GTCGAAATCTACGGACCGGAAT		

The primers for plasmid incompatibity group

Primer name	Sequence (5'-3')	product size (bp)
HI1-F	GGAGCGATGGATTACTTCAGTAC	471
HI1-R	TGCCGTTTCACCTCGTGAGTA	
HI2-F	TTTCTCCTGAGTCACCTGTTAACAC	644
HI2-R	GGCTCACTACCGTTGTCATCCT	
I1-F	CGAAAGCCGGACGGCAGAA	139
I1-R	TCGTCGTTCCGCCAAGTTCGT	
X-F	AACCTTAGAGGCTATTTAAGTTGCTGAT	376
X-R	TGAGAGTCAATTTTTATCTCATGTTTTAGC	
L/M-F	GGATGAAAACTATCAGCATCTGAAG	785
L/M-R	CTGCAGGGGCGATTCTTTAGG	
N-F	GTCTAACGAGCTTACCGAAG	559
N-R	GTTTCAACTCTGCCAAGTTC	
FIA-F	CCATGCTGGTTCTAGAGAAGGTG	462
FIA-R	GTATATCCTTACTGGCTTCCGCAG	
FIB-F	TCTGTTTATTCTTTTACTGTCCAC	702
FIB-R	CTCCCGTCGCTTCAGGGCATT	
W-F	CCTAAGAACAACAAAGCCCCCG	242
W-R	GGTGCGCGGCATAGAACCGT	
Y-F	AATTCAAACAACACTGTGCAGCCTG	765
Y-R	GCGAGAATGGACGATTACAAAACTTT	
P-F	CTATGGCCCTGCAAACGCGCCAGAAA	534

P-R	TCACGCGCCAGGGCGCAGCC	
FIC-F	GTGAACTGGCAGATGAGGAAGG	262
FIC-R	TTCTCCTCGTCGCCAAACTAGAT	
A/C-F	GAGAACCAAAGACAAAGACCTGGA	465
A/C-R	ACGACAAACCTGAATTGCCTCCTT	
T-F	TTGGCCTGTTTGTGCCTAAACCAT	750
T-R	CGTTGATTACACTTAGCTTTGGAC	
F_{repB}-F	TGATCGTTTAAGGAATTTTG	
F_{repB}-R	GAAGATCAGTCACACCATCC	
K/B-F	GCGGTCCGGAAAGCCAGAAAAC	160
K-R	TCTTTCACGAGCCCGCCAAA	
B/O R	TCTGCGTTCCGCCAAGTTCGA	159
FII-F	CTGATCGTTTAAGGAATTTT	270
FII-R	CACACCATCCTGCACTTA	

Primers used for PCR-mapping analysis of genetic environment

Primer name	Sequence (5'-3')	product size (bp)	Tm (℃)
IS26-F	GCACGCATCACCTCAATACC	Variable	56.7
fosA3-R2	TCATCCAGCGACAAGCACA		
fosA3-F2	GGGGCTGAGGTATGGAAAGA	Variable	56.1
IS26-R	AGGAGATGCTGGCTGAACG		
fosA-up1-F	ATACAGCATCGTCAGCTCGG	833	58
fosA-up1-R	CGTGCAGCTCCAGCTTGT		
fosA-up2-F	TGAACGCCCGCAAGCCAACAT	1313	65
fosA-up2-R	CCGCGACAACATCCCCGAAGTG		
fosA-d1-F	CTGGAATACCGGGGCCTATC	947	56
fosA-d1-R	TATCCTTGCCTCTCCGGTCA		
fosA-d2-F	GCGTCATATCGACGACGTTC	1543	58
fosA-d2-R	GCAGCCAGGTCTGGGTTTTA		
fosA-d3-F	GCTTACATGGCAAAGGTCG	1500	58
fosA-d3-R	ATCGCCGGCAACAAACAAAT		
fosA-d4-F	TCACCTCGTCGATGATTGCC	1044	57.5
fosA-d4-R	GTTGTTGATACCGCTCACGC		
IS26U	GGATAATCAACGCCACGCTG	Variable	57
ISEcp1U1	AAAAATGATTGAAAGGTGGT	Variable	52
CTX-M-R	CGATATCGTTGGTGGTGCCATA		
CTX-M-F	TTTGCGATGTGCAGTACCAGTAA	Variable	56
fosA3-2R	CGGTTATCTTTCCATACCTCAG		
fosA7-UP1-F	TACATGCCCTTCAACTACACCGT	1994	69
fosA7-UP1-R	GCTAAATCTCCCACATGCAGCTC		
fosA7-UP2-F	CATGCACTACGTACGGTTTTC	729	63

fosA7-UP2-R	TATGTGCAGCCATTATCAGCAG		
fosA7-D1-F	CTCTGAACCACTTAACGCTTG	1700	62
fosA7-D1-R	ATCGCCATAGATCTTCACCAG		
fosA7-D2-F	GAACCGTAATATCCCGTGCTT	594	61
fosA7-D2-R	CCTCTGGCAACGAATCATCAA		
fosA7-D3-F	CCGACCTTGCGATAATTCACC	1126	63
fosA7-D3-R	CAATACCATATGACGCCCGAT		
fosA7-D4-F	ACAATTGCGATCTTTTCCTGT	390	59
fosA7-D4-R	ATTACTGTGCTTTTCGTCACC		
